# Weekly dengue forecasts in Iquitos, Peru; San Juan, Puerto Rico; and Singapore

**DOI:** 10.1371/journal.pntd.0008710

**Published:** 2020-10-16

**Authors:** Corey M. Benedum, Kimberly M. Shea, Helen E. Jenkins, Louis Y. Kim, Natasha Markuzon

**Affiliations:** 1 Draper, Cambridge, Massachusetts, United States of America; 2 Department of Epidemiology, Boston University School of Public Health, Boston, Massachusetts, United States of America; 3 Department of Biostatistics, Boston University School of Public Health, Boston, Massachusetts, United States of America; University of Washington, UNITED STATES

## Abstract

**Background:**

Predictive models can serve as early warning systems and can be used to forecast future risk of various infectious diseases. Conventionally, regression and time series models are used to forecast dengue incidence, using dengue surveillance (e.g., case counts) and weather data. However, these models may be limited in terms of model assumptions and the number of predictors that can be included. Machine learning (ML) methods are designed to work with a large number of predictors and thus offer an appealing alternative. Here, we compared the performance of ML algorithms with that of regression models in predicting dengue cases and outbreaks from 4 to up to 12 weeks in advance. Many countries lack sufficient health surveillance infrastructure, as such we evaluated the contribution of dengue surveillance and weather data on the predictive power of these models.

**Methods:**

We developed ML, regression, and time series models to forecast weekly dengue case counts and outbreaks in Iquitos, Peru; San Juan, Puerto Rico; and Singapore from 1990–2016. Forecasts were generated using available weekly dengue surveillance, and weather data. We evaluated the agreement between model forecasts and actual dengue observations using Mean Absolute Error and Matthew’s Correlation Coefficient (MCC).

**Results:**

For near term predictions of weekly case counts and when using surveillance data, ML models had 21% and 33% less error than regression and time series models respectively. However, using weather data only, ML models did not demonstrate a practical advantage. When forecasting weekly dengue outbreaks 12 weeks in advance, ML models achieved a maximum MCC of 0.61.

**Conclusions:**

Our results identified 2 scenarios when ML models are advantageous over regression model: 1) predicting dengue weekly case counts 4 weeks ahead when dengue surveillance data are available and 2) predicting weekly dengue outbreaks 12 weeks ahead when dengue surveillance data are unavailable. Given the advantages of ML models, dengue early warning systems may be improved by the inclusion of these models.

## Author summary

Accurate and timely forecasts of dengue fever can help mitigate the impact of the disease. Currently, regression and time series models are frequently used to predict dengue cases and outbreaks. However, these models may be limited in terms of model assumptions and the number of predictors that can be included. Machine learning (ML) models offer an appealing alternative as they have a nonlinear framework and can be applied to high dimensional data. In this study, we compared the performance of ML algorithms with that of regression and time series models in predicting dengue cases and outbreaks from 4 to up to 12 weeks in advance in 3 dengue-endemic regions. Model predictions were based upon local dengue surveillance (e.g., case counts), population, temporal, and weather data. Many countries lack sufficient health surveillance infrastructure, as such we evaluated the contribution of dengue surveillance and weather data on the predictive power of the models. Our results identified 2 scenarios when ML models performed better than conventional models: 1) predicting dengue weekly case counts 4 weeks ahead when dengue surveillance data are available and 2) predicting weekly dengue outbreaks 12 weeks ahead when dengue surveillance data are unavailable. This research suggests that ML models can be a beneficial tool for dengue early warning systems.

## Introduction

Dengue fever, a mosquito-borne disease, poses a significant public health concern due to its re-emergence in tropical and sub-tropical regions [1]. In many countries where dengue is present, the disease is endemic. Globally, researchers estimate that dengue infects 390 million people per year [2]; however, only 50–100 million cases are detected due to the high asymptomatic rate [1–6]. Estimating dengue burden can be problematic due to delays in case identification, strong intra- and inter-annual variation in incidence, and the majority of cases being clinically mild or asymptomatic [7–10]. As a result, implementing effective vector control operations can be challenging [11]. To overcome these issues, the development of accurate and timely early warnings systems capable of predicting future dengue incidence that do not depend upon current dengue case data remains an active area of research [5].

Several modeling approaches have been evaluated as early warning models for various infectious diseases. Time series and regression models are commonly used but have had various levels of success [5,7,12–28]. These models offer a robust and easily interpretable framework; however, these approaches can be limited by the underlying model assumptions (e.g., linear relationships between predictors and outcome) and the number of predictors that can be included [29,30]. Mechanistic models, which model individual components of a dynamic system, have accurately described outbreaks of influenza and mosquito borne diseases [31–37]; yet, the data required to parameterize these models are difficult to obtain, and the necessary model assumptions (e.g., disease infectivity) may not be clear until after the outbreak [7]. Ensemble approaches, which integrate multiple forecasting methods, have performed well and lately have received increased interest. Using dengue and climate data from Iquitos and San Juan, Buczak et al. [38] developed an ensemble of 300 models, which included Method of Analogs and Holt-Winters models, to predict various characteristics of dengue outbreaks (e.g., peak week, peak week incidence, and total cases in a season). The approach employed by Buczak et al. performed well when predicting peak week and total number of cases in a season but had difficulty predicting when the peak week would occur [38]. Yamana et al. [39] integrated multiple models, including a mechanistic model among others, to forecast dengue incidence in San Juan. In this study, the authors used Bayesian model averaging to integrate model results and found that the ensemble approach outperformed each of the individual models.

In contrast to the previously described approaches, machine learning (ML) models offer an appealing alternative and have already been used to successfully predict infectious disease case counts and outbreaks [40–46]. Similar to mechanistic models, ML models have a nonparametric and nonlinear modeling structure, but unlike regression and mechanistic models, ML approaches are independent of *a priori* specification of variable relationships, and can accommodate high dimensional data. Additionally, several ML models employ an ensemble framework to improve model accuracy. Though ML models have demonstrated good accuracy, the performance of these models have typically not been compared with the performance of more conventional approaches [45].

Regardless of the selected statistical framework, dengue prediction models typically use 2 types of inputs–a measure of prior dengue case counts and local weather conditions [7]. Prior dengue cases counts are included because there is strong relationship between current and subsequent levels of dengue, given the infectious nature of the disease. A weather component is included to describe how short-term changes in atmospheric conditions affect dengue vectors, hosts, and the infectious agent itself. In the case of dengue, rainfall plays an integral role in creating suitable breeding conditions for its vector, the *Aedes* mosquito [47–49]. Temperature also is known to affect larvae development, adult biting behavior, and the replication rate of the dengue virus [4,47,50–53]. Likewise, humidity improves egg longevity by preventing environmental desiccation [54–56].

In this study, we developed models using dengue surveillance (e.g., case counts), population, and weather data from 3 dengue-endemic locations to predict dengue case counts and outbreaks (i.e., where the number of reported cases exceeded a predefined threshold) 4 to 12 weeks in advance. We selected these 2 outcomes because case counts are an objective prediction measure where uncertainty can be easily quantified, while weekly outbreak occurrence is more relevant within the context of public health decision making [7]. We used forecast horizons of 4 and up to 12 weeks to develop models that can provide real-time updates and to provide timely warnings to give governmental authorities adequate response time, respectively [11]. We then used these models to examine 3 questions: (1) “*How well do ML models (i*.*e*., *Random Forest [RF] and Random Forest-Univaraite Flagging Algorith [RF-UFA]) forecast dengue*, *relative to commonly used prediction models (i*.*e*., *Poisson regression*, *Logistic regression and autoregressive integrated moving average [ARIMA] models)*?” (2) “*How is model accuracy impacted by the availability–or lack of–current dengue surveillance data*?” and (3) “*Among data used in our models*, *what were the strongest predictors of the weekly number of reported dengue cases*?”

## Materials and methods

### Study areas

We predicted dengue case counts and outbreaks in 3 endemic locations: Iquitos, Peru; San Juan, Puerto Rico; and Singapore. Iquitos is a geographically isolated port city located on the Amazon River with a population of approximately 400,000 people [57,58]. Rainfall occurs year round and is heaviest between November and May. The mean daily temperatures of the coolest and hottest months are 25.6°C and 27.5°C, respectively. San Juan is the capital and largest city in Puerto Rico. It is located on the Northeastern coast of the island, and has an approximate population of 400,000 people. Rainfall primarily occurs between April and November, leaving the other months relatively dry. The mean daily temperatures of the coolest and hottest months are 25.3°C and 28.7°C, respectively. Singapore is a city state off the Southern-most tip of the Malay Peninsula, and has approximately 5.6 million inhabitants. Rainfall is heaviest during the Northeast monsoon season, which typically occurs from November to March [59]. A second drier monsoonal period occurs between June and October. The mean daily temperatures of the coolest and hottest months in Singapore are 26.5°C and 28.4°C, respectively.

### Dengue surveillance data, predictors, and outcomes

Weekly dengue case counts for Iquitos were available between June 2000 and June 2013 from a passive surveillance network representing approximately 40% of the Iquitos population [57,60,61]. Weekly case counts for San Juan were available from April 1990 to April 2013 and were ascertained from a combination of active and passive surveillance systems [62]. All confirmed dengue cases, regardless of severity were reported in Iquitos and San Juan. Further, when the number of samples exceeded local testing capactiy, the number of positive cases among those not tested was estimated by multiplying the number of untested cases by the rate of laboratory-positive cases amongst those that were tested [57,60,62]. In both locations, all dengue and DHF cases were reported together. For Singapore, weekly dengue and DHF cases [63] for were reported separately and available between January 2000 and December 2016 from the Ministry of Health. Dengue is a nationally notifiable disease in Singapore, meaning that all clinically diagnosed and laboratory-confirmed cases must be reported to the Ministry of Health within 24 hours [28,63]. Clinically confirmed cases were then confirmed with serologic or virologic testing by the Ministry of Health. Data from each of the 3 study locations are publically available [61,64].

Using weekly case counts, we created surveillance-based predictors for our models (S1 Table). We summarized observed dengue case counts with weekly and cumulative totals starting from the beginning of the year. We also summarized the annual number of dengue cases in the past 1 to 3 years.

These data also served as the prediction outcomes, “*weekly case counts*” and “*weekly outbreaks*.” We created the binary outcome variable, weekly outbreaks, to indicate whether or not weekly case counts exceeded a predefined threshold. For this study, the outbreak threshold was set at 1.5 standard deviations above the mean weekly reported cases and is defined as:

$${Weekly\;Outbreak}_{W}=\left\{ \begin{array}{c} 1,{Cases}_{W}\geq Outbreak\;threshold\\ 0,Otherwise \end{array}\right.$$ (1)

where Cases_T_ is the number of reported dengue cases for week “*T*” (the week of interest). The outbreak threshold was defined as:

$$Outbreak\;threshold={\bar{Cases}}_{training}+1.5*\sqrt{\frac{\sum_{W=1}^{n}{\left({Cases}_{T,training}-{\bar{Cases}}_{training}\right)}^{2}}{n-1}}$$ (2)

where Cases_T,training_ is the number of cases reported for week *T* in the training data (a subset of the study data, including outcome [e.g,. weekly outbreaks] and predictor variables [e.g., 7-day average temperature, 7-day average absolute humidity], used to develop the predictive model and is discussed in more detail in section *Prediction Approach*); ${\bar{Cases}}_{training}$ is the average weekly case counts in the training data; and *n* is the number of observed weeks in the training data.

### Population data and predictors

We used government data to generate population estimates for each study area. Population estimates for the Iquitos metropolitan area (2000–2014) and the San Juan-Carolina-Caguas Metropolitan Statistical Area (1990, 1999–2014) came from the National Statistical Institute of Peru and the U.S. Census Bureau, respectively [61]. Population estimates for Singapore (2000–2016) were obtained from the Ministry of Trade and Industry, Department of Statistics and are derived from registry-based administrative data [65,66]. For years without population estimates, we imputed the missing data with a linear regression model where total population was regressed by year.

Singapore is unique in that it has a highly mobile population with large influxes of travelers. To account for the variation in nonresidents, we identified government data detailing the monthly number of air passenger arrivals at Changi Airport (1999–2016) [67]. With these population and air travel data we created additional predictor variables for our models (S2 Table).

### Temporal predictors

Inter- and intra-annual variations in dengue cases have been observed across the globe, providing evidence for multi-year periodicity which has been estimated to be approximately 3 years [57,68–70]. To account for the temporal variation in dengue cases, we summarized time by including the month that the week of interest occurs in and 1 to 4 year periodic components as predictor variables (S2 Table). The periodic components were sine and cosine functions described below:

$$sin\;(\frac{2\pi t}{12*a})$$ (3)

$$cos\;(\frac{2\pi t}{12*a})$$ (4)

where *t* is the number of months since the start of the study period and *a* is the inter-annual period length in years.

### Weather data and predictors

We ascertained daily temperature, humidity, and rainfall summaries (i.e., averages, minimums, maximums, and totals) from the National Oceanic and Atmospheric Administration and the National Environment Agency, Singapore (Table 1). We obtained weather measurements from weather stations, remote sensed imagery, and meteorological reanalysis to account for the various strengths and limitations of each data source (see Weather data limitations in Supplemental S1 Text for a brief overview of these limitations) [12,71–77]. Daily weather summaries obtained from remote sensed imagery and meteorological reanalysis were collected from the gridded cell surrounding the weather station used for each study area. We collected daily weather summaries from January 1999 to March 2014 for Iquitos, January 1989 to April 2013 for San Juan, and January 1999 to December 2016 for Singapore.

**Table 1:** Weather variables obtained by data source.

Variable	Units	Resolution	Source
Daily minimum air temperature	Celsius	NA	Weather Station
Daily average air temperature	Celsius	NA	Weather Station
Daily maximum air temperature	Celsius	NA	Weather Station
Daily diurnal air temperature range	Celsius	NA	Weather Station
Daily total rainfall	millimeters	NA	Weather Station
Daily total rainfall	millimeters	0.25x0.25 degrees	Remote Sensed
Daily absolute humidity	g/m^3^	0.5x0.5 degrees	Meteorological Reanalysis
Daily relative humidity	%	0.5x0.5 degrees	Meteorological Reanalysis
Daily specific humidity	g/kg	0.5x0.5 degrees	Meteorological Reanalysis
Daily dew point	Kelvin	0.5x0.5 degrees	Meteorological Reanalysis
Daily minimum air temperature	Kelvin	0.5x0.5 degrees	Meteorological Reanalysis
Daily average air temperature	Kelvin	0.5x0.5 degrees	Meteorological Reanalysis
Daily maximum air temperature	Kelvin	0.5x0.5 degrees	Meteorological Reanalysis
Daily diurnal air temperature range	Kelvin	0.5x0.5 degrees	Meteorological Reanalysis
Daily average surface temperature	Kelvin	0.5x0.5 degrees	Meteorological Reanalysis
Daily Total Rainfall	Kelvin	0.5x0.5 degrees	Meteorological Reanalysis

* Temperature measurements reported in Kelvin were converted to Celsius.

We created weather-based predictors for our models (S3 Table) by aggregating daily weather summaries into multi-day and multi-week summaries. Temperature and humidity predictors included 7-, 14-, 21-, and 28-day moving averages and standard deviations. As temperature alone does not account for the optimal temperature ranges for the Aedes mosquito and may not accurately represent the temperature-dengue relationship, we created additional temperature predictors based upon the Temperature Suitability Index (TSI) [78]. Rainfall predictors included 7-, 14-, 21-, and 28-day moving averages, standard deviations, and total number of days with any recorded rainfall. We also summarized daily total rainfall for cumulative periods of 1- to 20-weeks. Since the effect of rainfall on mosquito abundance has been found to differ across seasons [70,79] we created additional rainfall predictors that summarized daily total rainfall for cold, warm, and hot periods which were based upon average daily temperature and the extreme minimum and maximum TSI thresholds [70,78].

### Missing weather data

We observed missing daily weather measurements in each area due to non-reporting or instrument failure (S1 Fig). We imputed missing weather data using multiple imputation by chained equations with the MICE R package [80]. For this study, we created 10 imputation sets which we then averaged to obtain a final value for each missing observation [81].

### Prediction approach

**Fig 1:**
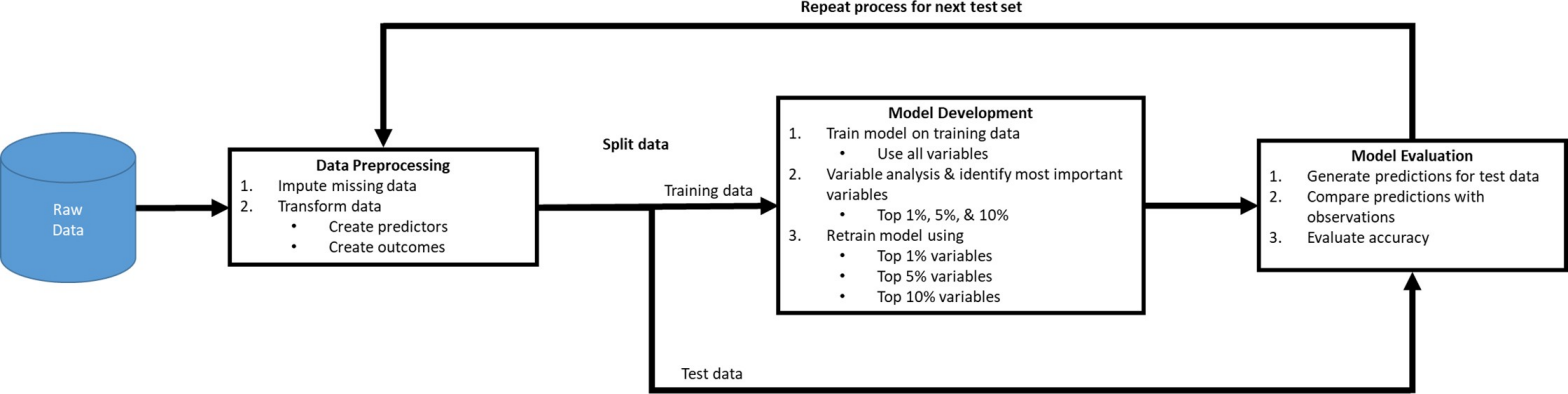
General framework to develop RF and regression prediction models. To assess how each model’s predictive accuracy was affected by the lack of current dengue surveillance data, we trained models to predict dengue case counts and outbreaks using only population, temporal, and weather predictor variables. We compared the performance of these models with the performance of the same models when surveillance data inputs were included.

In our analysis, we developed models to predict dengue case counts and outbreaks based upon the temporal variation in dengue activity, regional population, and weather. Fig 1 reflects the general framework, used in this study, for developing a predictive ML (i.e., RF, RF-UFA) and regression-based models (i.e., Poisson regression, Logistic Regression) using historical and near-real time data as input. In our approach, we trained (i.e., fit to data) models with a subset of the study data (i.e., training data) and evaluated the accuracy of model forecasts on the last 4 years’ worth of data (i.e., testing data) that had been withheld during model training. We evaluated each model on 1 year’s worth of testing data at a time and in chronological order. After model evaluation, the test set was then added to the training data and the process was repeated for the subsequent year of test data. This resulted in each model being redeveloped and retrained for each year of testing data. Each model made 4 and 12 week prospective forecasts from week “*T*” using the previous 26 weeks of predictor data (*T-1*, *T-2*, …, *T-26*).

For each trained ML and regression model, we analyzed the predictor variables and assessed their importance. The variable analysis allowed us to (1) identify the strongest predictors of dengue case counts for each study area and (2) to perform variable reduction, a conventional approach to improve model accuracy. During variable reduction, we removed weak and non-informative predictors by ranking each variable according to the variables measure of importance, which is defined later. After ranking each variable, we removed all non-informative variables and selected the top 1%, 5%, and 10% most important variables. We then retrained each model using the 3 subsets of predictors and evaluated the predictive accuracy of these models. This process was performed for each test set.

For this study, all models and statistical analyses were implemented in the R programming environment version 3.3.3.[82]

### Predicting weekly outbreaks

We observed substantial imbalance in the proportion of outbreak and non-outbreak weeks for each study area. Class imbalance can cause a predictive model to classify all predictions as the same class in an effort to maximize model accuracy, resulting in an uninformative model. To overcome the limitation of class imbalance [83], we trained the models on a “balanced” dataset where we under-sampled non-outbreak observations to create a 1:1 ratio of outbreak to non-outbreak observations in the training set. To account for sampling variability, we created 500 training sets which we used to train each model and averaged the predictions. Additionally, we optimized model performance by selecting the classification threshold (i.e., the minimum prediction value required for an observation to be classified as “*outbreak*”) that maximized model performance.

### Machine learning models

In our study we used RF to predict weekly case counts and weekly outbreaks and RF-UFA to predict weekly outbreaks only. RF is an ensemble ML algorithm based upon decision trees and has been previously used to analyze time series data [40,45,84]. RF-UFA is an extension of the RF algorithm where the Univariate Flagging Algorithm (UFA) is used to transform continuous predictors into binary predictors [85]. UFA transforms continuous predictors by identifying an optimal threshold that is associated with a statistically significant (p ≤ 0.01) higher (“high-risk”) or lower (“low-risk”) risk of the outcome. All RF models were fitted with the *randomForest* R package [86]. A more detailed explaination of both models is available in Supplemental S1 Text *“Overview of machine learning models*.*”*

### Regression models

We used 2 types of generalized linear regression models in our study: Poisson regression to predict weekly case counts and Logistic regression to predict weekly outbeaks. Unlike RF, regression models are not well suited for high dimensional data analysis and requires additional measures to prevent overfitting. To minimize this risk, we used the Least Absolute Shrinkage and Selection Operator (LASSO) algorithm [87–89]. We identified the optimal penalty parameter using 10-fold cross validation and selecting the parameter that minimized the cross validation mean absolute error (MAE), for Poisson regression models, and the misclassification error rate, for Logistic regression models [89]. All Poisson regression and Logistic regression models were implemented with the *glmnet* R package [90].

### Time series model

We developed an autoregressive integrated moving average (ARIMA) model to forecast weekly dengue case counts in each study location. As ARIMA models cannot be applied to high dimensional data, model predictions were based upon the time series of observed case counts only. In this study, we also evaluated seasonal ARIMA (SARIMA) models and found that the added seasonal component did not consistently improve model performance, as such we do not present the results of the SARIMA model.

The ARIMA model parameters were identified by finding the parameters that resulted in the best fit of the training data. To identify the best fitting parameters, we performed a stepwise search and selected the parameters which minimized the model Akaike Information Criterion (AIC). The ARIMA model was implemented using the *forecast R* package [91].

### Variable importance

Variable importance is a measure of how much a single variable contributes to the overall predictive accuracy of a model. For RF-based models, we ranked variables according to their “*percentage increase in mean squared error*” when predicting weekly case counts and by their “*mean decrease in accuracy*” when predicting weekly outbreaks [92]. Both metrics measure how much error would be introduced into the model’s predictions if the variable were to be removed from the model. For Poisson regression and Logistic regression, we ranked variables according to the absolute value of the standardized coefficient, a conventional ranking approach for regression models [93].

### Model evaluation

We evaluated the performance of each model with the withheld testing data. To quantify model accuracy, we selected accuracy metrics that measure how well model predictions approximate observed outcomes. When predicting weekly case counts, we used mean absolute error (MAE) which measures how far a prediction deviates from the observed outcome. The MAE is defined as follows:

$$MAE=\frac{1}{n}\sum_{i=1}^{n}\left|y_{i}-{\hat{y}}_{i}\right|$$ (5)

where *n* is the number of observations, *y_i_* is the observed number of dengue cases for week *i*, and ${\hat{y}}_{i}$ is the predicted number of dengue cases for week *i*. The MAE is considered to be an unbiased estimator because it only considers the variance and not the magnitude of the errors [45]. Since the magnitude of reported dengue cases varied widely by study area, we also report the normalized MAE (nMAE). The nMAE provides an estimate of the prediction error relative to the average number of weekly cases in the testing data and allows for better comparisons of model accuracy between study areas and forecast horizons. We calculated the nMAE by dividing the MAE by the average weekly number of dengue cases. The nMAE is defined as follows:

$$nMAE=\frac{MAE}{\frac{1}{n}\sum_{i=1}^{n}y_{i}}$$ (6)

where *n* is the number of observations *y_i_* is the observed number of dengue cases for week *i*, and *MAE* is the mean absolute error. The best value that can be obtained for both MAE and nMAE is 0, while the worse value is unbounded.

For models forecasting weekly outbreaks, we quantified how well model predictions approximated observed outcomes with Matthew’s Correlation Coefficient (MCC) [94]. MCC measures the correlation between a binary outcome and prediction and unlike other measures MCC is insensitive to class imbalance [95,96]. MCC is defined as follows:

$$MCC=\frac{TP*TN-FP*FN}{\sqrt{\left(TP+FP)*(TP+FN)*(TN+FP)*(TN+FN\right)}}$$ (7)

where *TP* is the number of true positives; *TN* is the number of true negatives; *FP* is the number of false positives; and *FN* is the number of false negatives. The best value that can be obtained for MCC is +1, while the worse value is -1.

## Results

**Fig 2:**
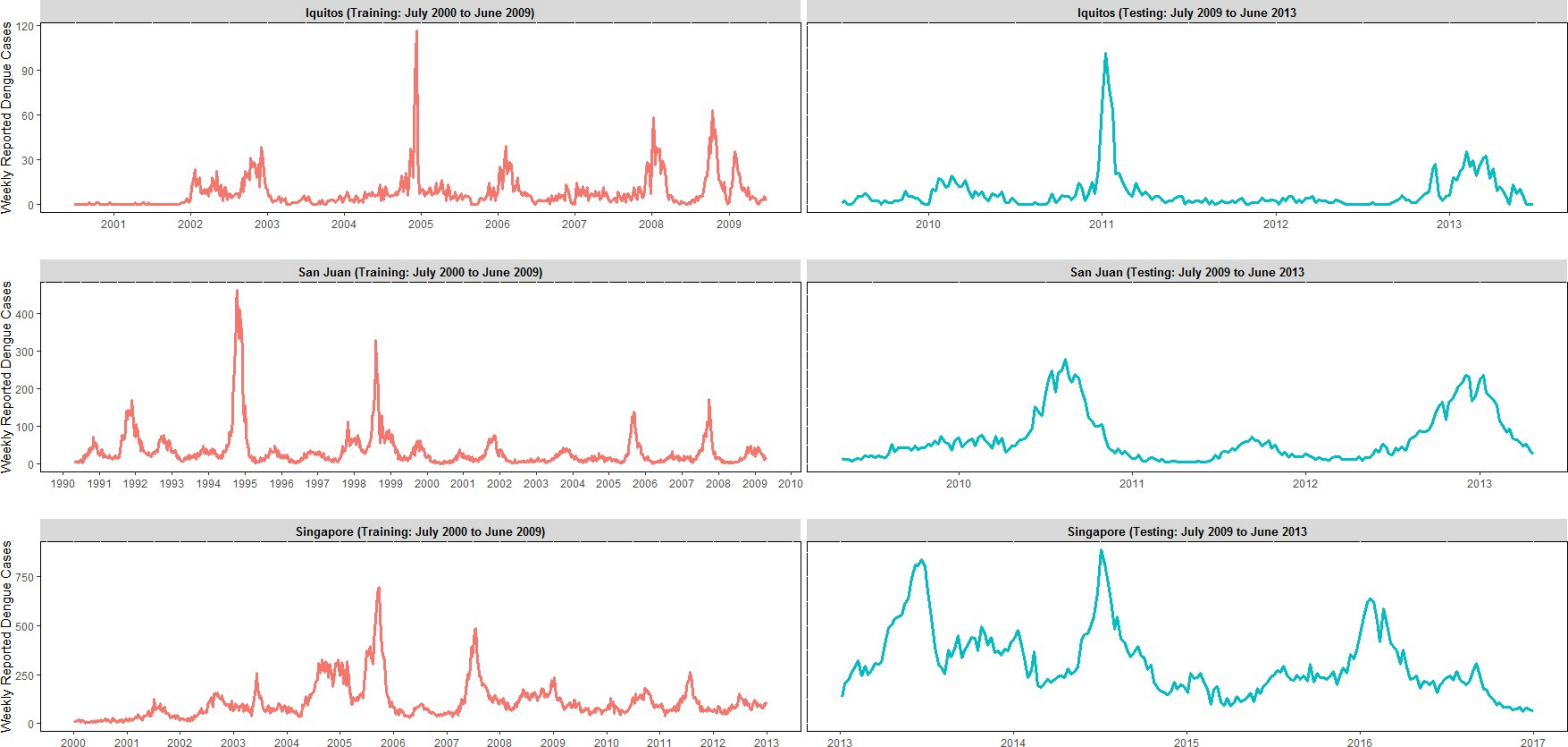
Weekly observations of reported dengue cases by study area. In this figure, left-hand panels (red curves) represent training data, while right-hand panels (blue curves) represent the testing data.

Weekly dengue case counts for each study area are presented in Fig 2. We observed substantial inter-annual variation as well as wide ranges in the number of weekly reported cases during the observational periods by study area. Reported weekly case counts ranged from 0 to 116 in Iquitos, 0 to 461 in San Juan, and 3 to 888 in Singapore. The average number of weekly cases varied greatly by study area as well. The average number of weekly cases was 7.57, 38.84, and 115.96 for Iquitos, San Juan, and Singapore, respectively. In 2013, we observed a notable increase in the number of reported dengue cases in Singapore, which was the result of a large dengue outbreak throughout all of Southeast Asia [97–100].

In our study, we developed multiple ML, regression-based, and time series models under various data availability and forecast horizon settings. Since the objective of this study was to compare ML (i.e., RF and RF-UFA) models with conventional forecasting models, we only describe the results for models with the best performance under each data-forecast horizon scenario. In our evaluation, models with the smallest nMAE or largest MCC were defined as the best performing models.

### Forecasting dengue case counts

**Fig 3:**
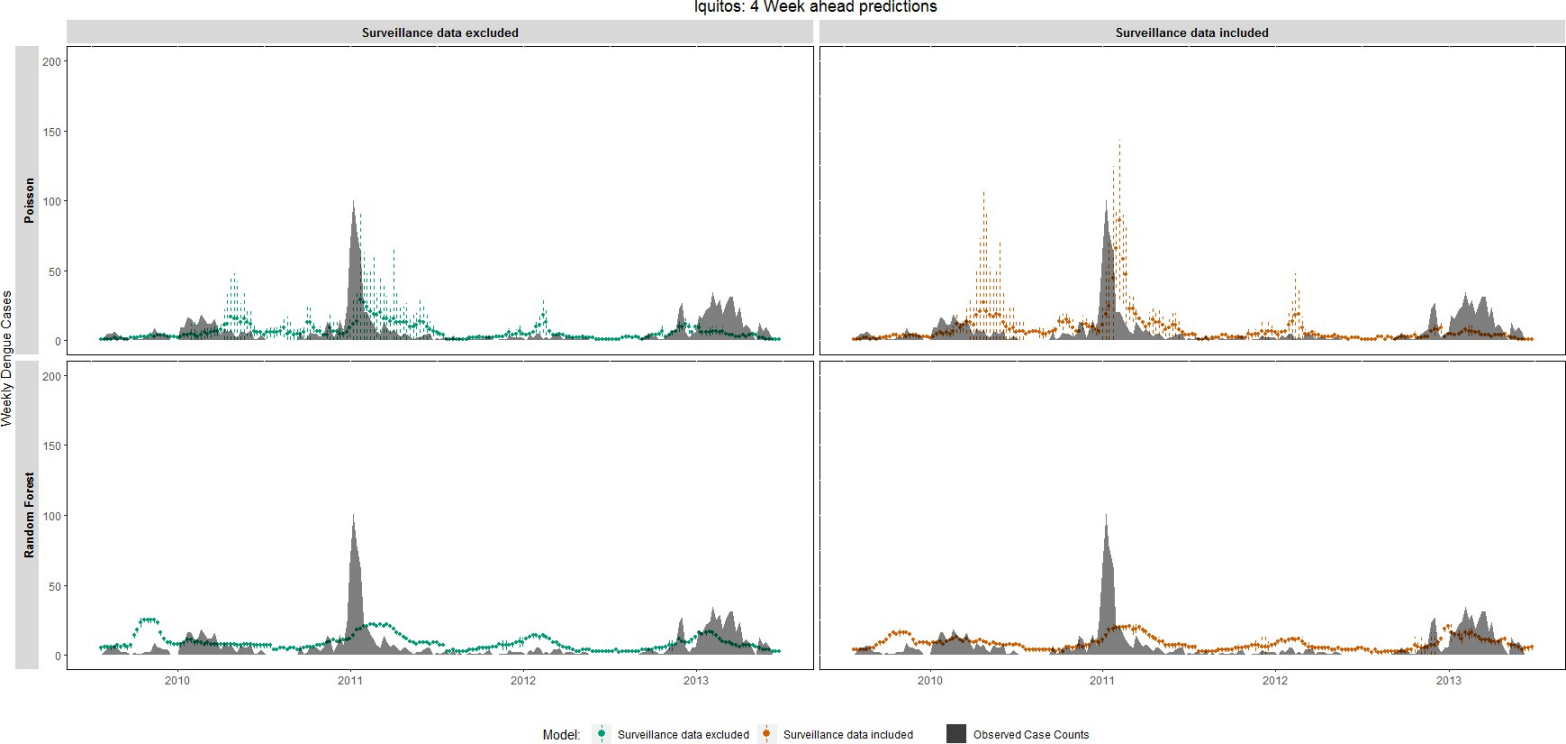
4 week forecast accuracy of the temporal pattern of dengue case counts, Iquitos, Peru, June 2009 –June 2013. Observed weekly cases counts (black area) are compared with 4 week ahead forecasts made by Random Forest and Poisson regression models. Dotted lines represent 95% confidence intervals around the model’s prediction. RF model standard errors were estimated using the infinitesimal jackknife for bagging approach [101].

In Iquitos (4 week ahead forecasts: Fig 3; 12 week ahead forecasts: S2 Fig), both RF and Poisson regression models did not fully capture the sharp increase in dengue cases in 2011. Interestingly, during the typical peak dengue period the predictions made by the Poisson regression model had the highest level of uncertainty as demonstrated by the wide confidence intervals. Unlike the Poisson regression model’s predictions, RF model forecasts had small confidence intervals regardless of the transmission period (peak or non-peak season). Forecasts made by ARIMA model (S3 Fig) typically captured the transmission dynamics (i.e., increased cases during the peak season and fewer cases during the low dengue season); however, ARIMA model forecasts did not marginally vary from year to year, indicating an inability to differentiate between large and small epidemics.

**Fig 4:**
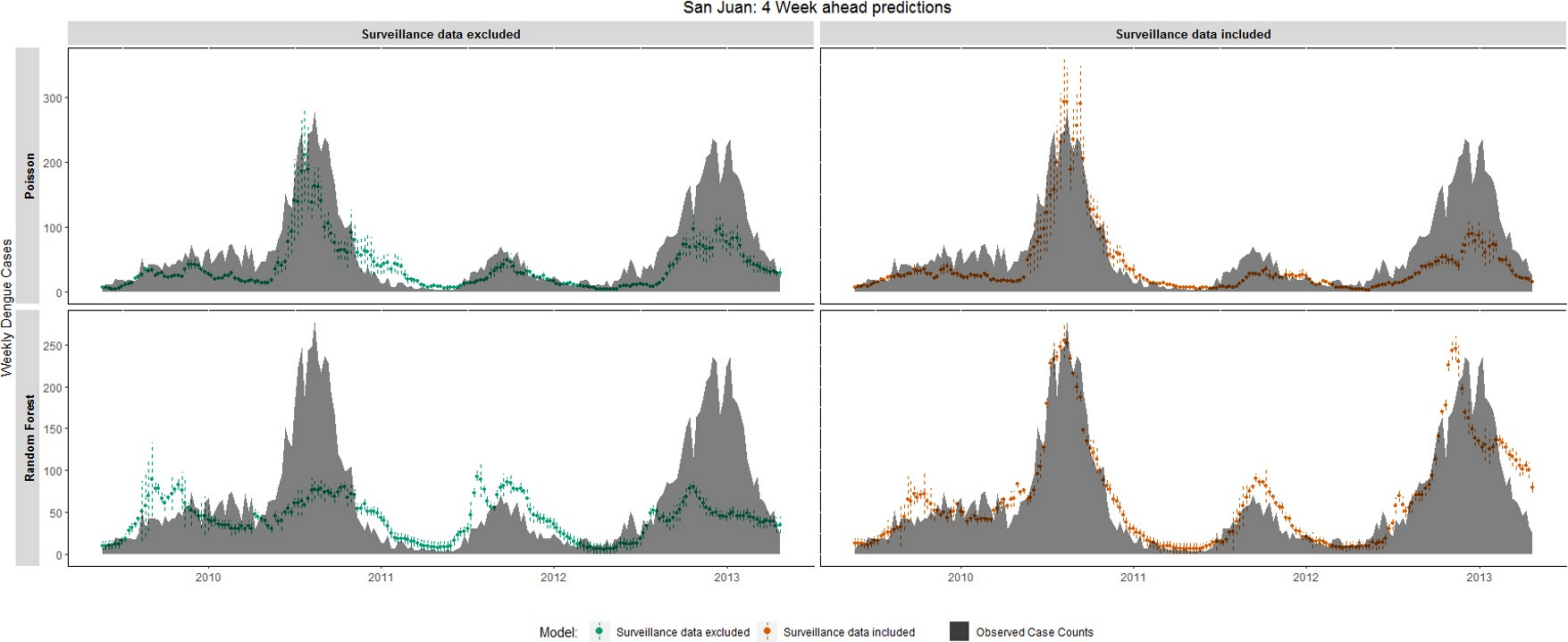
4 week forecast accuracy of the temporal pattern of dengue case counts, San Juan, Puerto Rico, April 2009 –April 2013. Observed weekly cases counts (black area) are compared with 4 week ahead forecasts made by Random Forest and Poisson regression models. Dotted lines represent 95% confidence intervals around the model’s prediction. RF model standard errors were estimated using the infinitesimal jackknife for bagging approach [101].

In San Juan, both RF and Poisson models captured the general trend in dengue case counts regardless of the inclusion of surveillance data (Fig 4). When surveillance data were included, both RF and Poisson model forecasts were more similar to observed case counts as when surveillance data were not included. As was observed in Iquitos, Poisson model forecasts showed higher levels of uncertainty, especially during the peak dengue period. Confidence intervals around RF model forecasts remained consistent throughout the testing period. When forecasting dengue cases 12 weeks in advance (S4 Fig), RF and Poisson regression models again reflected the general trends in dengue cases. Similarly, the ARIMA model (S5 Fig) at times captured the general dynamics; however, there were several occasions where the model predicted increases in dengue cases several weeks after the observed peak week.

**Fig 5:**
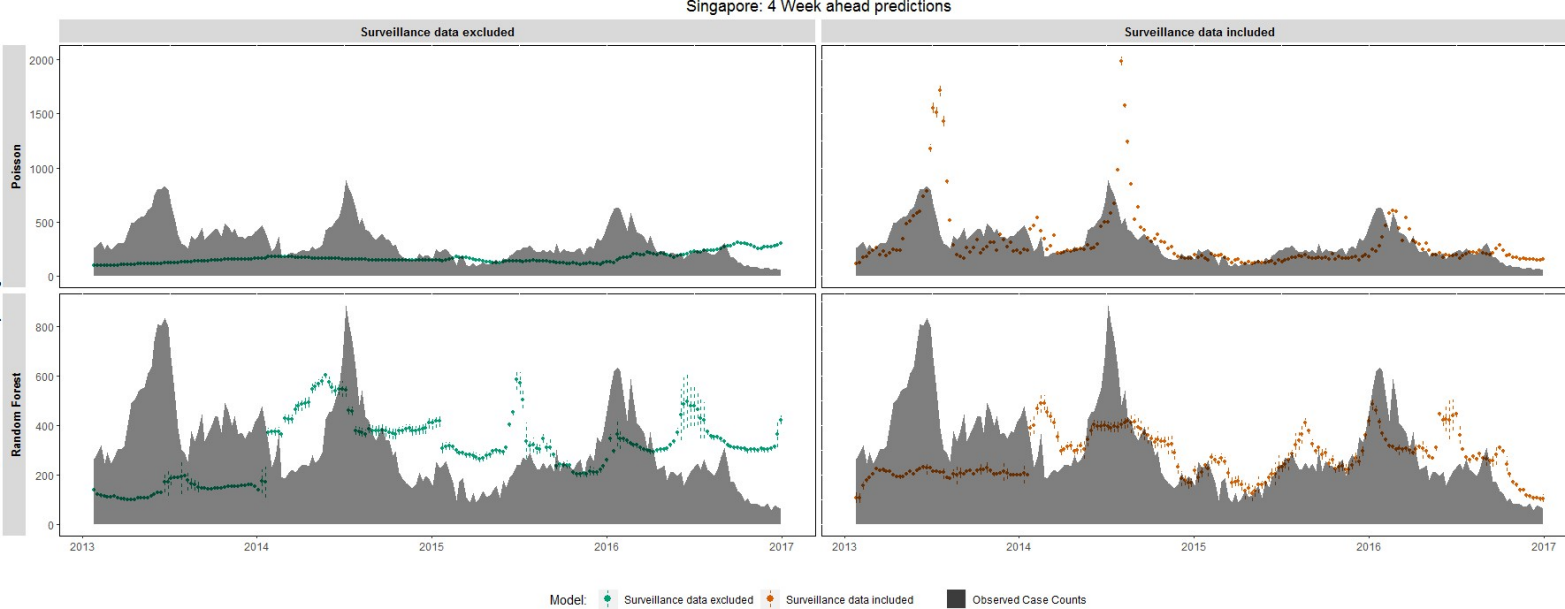
4 week forecast accuracy of the temporal pattern of dengue case counts, Singapore, January 2013 –December 2016. Observed weekly cases counts (black area) are compared with 4 week ahead forecasts made by Random Forest and Poisson regression models. Dotted lines represent 95% confidence intervals around the model’s prediction. RF model standard errors were estimated using the infinitesimal jackknife for bagging approach [101].

In Singapore (Fig 5), when surveillance data were included in the model, RF and Poisson regression 4 week ahead predictions did not reflect the general trend in dengue cases for the first 2 sets of testing data (2013 and 2014). In the last 2 test sets (2015 and 2016) 4 week ahead forecasts for both RF and Poisson regression captured the general trend in dengue cases, suggesting that the training data was not representative of the first 2 test sets (2013 and 2014). A similar trend was also observed for the ARIMA model (S6 Fig) where the model was unable to capture the general trend in the first 2 test sets (2013 and 2014) but improved in the last 2 test sets (2015 and 2016). When surveillance data were removed, both models performed poorly. When model forecasts were extended to 12 weeks in advance (S7 Fig), both RF and Poisson regression performed poorly, even when the model inputs included surveillance data. Similarly, the ARIMA model’s 12 week ahead forecasts did not reflect the general trend in dengue cases.

Table 2 summarizes the nMAE and MAE of the residuals between observed weekly dengue case counts and model predictions for the optimal RF, Poisson regression, and ARIMA models by study area and the data used to make the predictions (results for all evaluated models are available in S4 Table). When the evaluated models predicted dengue cases 4 weeks ahead and surveillance data were included, RF had more accurate forecasts relative to both Poisson regression and ARIMA models. We estimated RF nMAEs as 0.87, 0.27, and 0.40 in Iquitos, San Juan, and Singapore respectively. On average, RF forecasts had 21% and 33% less error than Poisson regression and ARIMA models. As model performance may differ by dengue season, we also evaluated model accuracy during peak and non-peak dengue periods [102–104]. During peak dengue season (S5 Table), the RF model had less error than Poisson regression and ARIMA models in San Juan (RF nMAE: 0.22) and Singapore (RF nMAE: 0.37). In Iquitos, the ARIMA model had the least amount of error (ARIMA nMAE: 0.70). During the non-peak dengue (S6 Table), Poisson regression had the least amount of error in Iquitos (Poisson nMAE: 0.91) while RF had the smallest nMAE in San Juan (RF nMAE: 0.37). In Singapore, RF and Poisson regression had identical nMAEs, 0.43.

**Table 2:** Optimal model performance when predicting weekly dengue case counts.

	4 weeks ahead forecast accuracy			12 weeks ahead forecast accuracy	
	Iquitos	San Juan	Singapore	San Juan	Singapore
	nMAE (MAE)	nMAE (MAE)	nMAE (MAE)	nMAE (MAE)	nMAE (MAE)
**Surveillance Data Included**					
Random Forest	0.87 (6.26)	0.27 (17.53)	0.4 (126.12)	0.48 (32.46)	0.62 (192.76)
Poisson Regression	1.02 (7.30)	0.45 (29.41)	0.44 (135.98)	0.59 (39.50)	0.66 (205.65)
ARIMA*	0.94 (6.75)	0.67 (43.52)	0.58 (182.34)	1.16 (78.41)	0.40 (126.34)
**Surveillance Data Excluded**					
Random Forest	0.96 (6.89)	0.59 (38.31)	0.61 (190.00)	0.57 (38.46)	0.62 (193.09)
Poisson Regression	0.88 (6.31)	0.50 (32.63)	0.58 (181.17)	0.56 (37.51)	0.65 (204.35)

* The ARIMA model was only developed using previously observed case counts, as such results are not shown when surveillance data are excluded for this model.

Abbreviations: nMAE: normalized mean absolute error; MAE: mean absolute error.

Results for all evaluated models are available in S4 Table.

We evaluated each model’s ability to make long-term forecasts of dengue case counts. Compared with RF and Poisson regression, ARIMA had a smaller nMAE in Iquitos and Singapore, 0.85 and 0.40 respectively. However, in San Juan, RF (nMAE: 0.48) had less error than Poisson regression (nMAE: 0.59) and ARIMA (nMAE: 1.16). We observed similar trends in performance during the peak-dengue season (S5 Table). During non-peak dengue season (S6 Table) RF was more accurate than Poisson regression and ARIMA in Iquitos and San Juan (Iquitos RF nMAE: 1.34; San Juan RF nMAE: 0.59). In Singapore, ARIMA performed better than both RF and Poisson regression (ARIMA nMAE: 0.43).

To understand how model accuracy is affected when current surveillance data are unavailable, we retrained models using only population, temporal, and weather data inputs. We found that for near term forecasts RF nMAEs were equal to 0.96, 0.59, and 0.61 in Iquitos, San Juan and Singapore respectively. We observed Poisson regression nMAEs equal to 0.88, 0.50 and 0.58 in Iquitos, San Juan, and Singapore. During peak dengue season RF had the least amount of error in Iquitos (RF: 0.80; Poisson: 0.89) and Singapore (RF: 0.57; Poisson: 0.62) but more error than the Poisson regression model in San Juan (RF: 0.58; Poisson: 0.45). During the non-peak season, the Poisson regression model had smaller or similar nMAEs (Poisson Iquitos: 0.85; San Juan: 0.59; Singapore: 0.55) relative to RF (RF Iquitos: 1.36; San Juan: 0.59; Singapore: 0.63).

For long-term forecasts in Iquitos and San Juan, the RF model (nMAE = 0.96 and 0.57 respectively) was less accurate than Poisson regression; we estimated Poisson regression nMAEs as 0.87 and 0.56 for Iquitos and San Juan respectively. In Singapore, we estimated RF and Poisson regression nMAEs as 0.62 and 0.65, indicating similar model accuracy.

### The strongest predictors of dengue case counts

**Fig 6:**
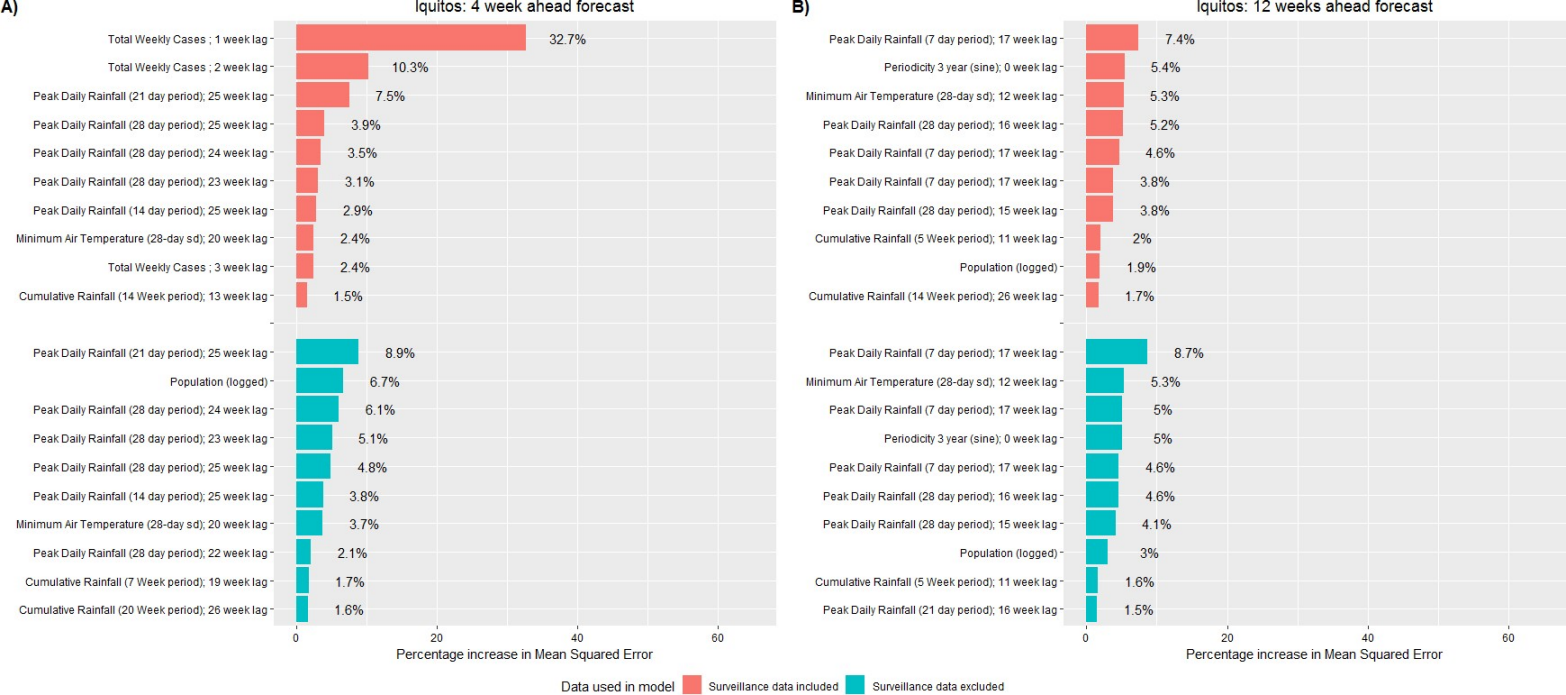
Top 10 most important predictors for the Random Forest model when predicting weekly dengue case counts, Iquitos, Peru. The 10 most important predictors to the Random Forest model prior to variable reduction. Predictor importance was quantified as the percentage increase in mean squared error. Red bars indicate the model included surveillance data inputs while blue bars indicate the model did not include surveillance data inputs. Predictors are shown for forecasts made 4 (A) and 12 (B) weeks in advance.

**Fig 7:**
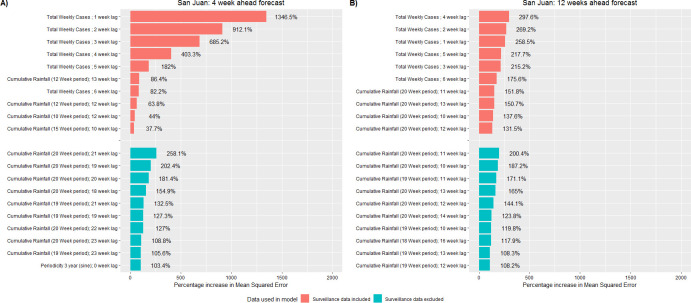
Top 10 most important predictors for the Random Forest model when predicting weekly dengue case counts, San Juan, Puerto Rico. The 10 most important predictors to the Random Forest model prior to variable reduction. Predictor importance was quantified as the percentage increase in mean squared error. Red bars indicate the model included surveillance data inputs while blue bars indicate the model did not include surveillance data inputs. Predictors are shown for forecasts made 4 (A) and 12 (B) weeks in advance.

**Fig 8:**
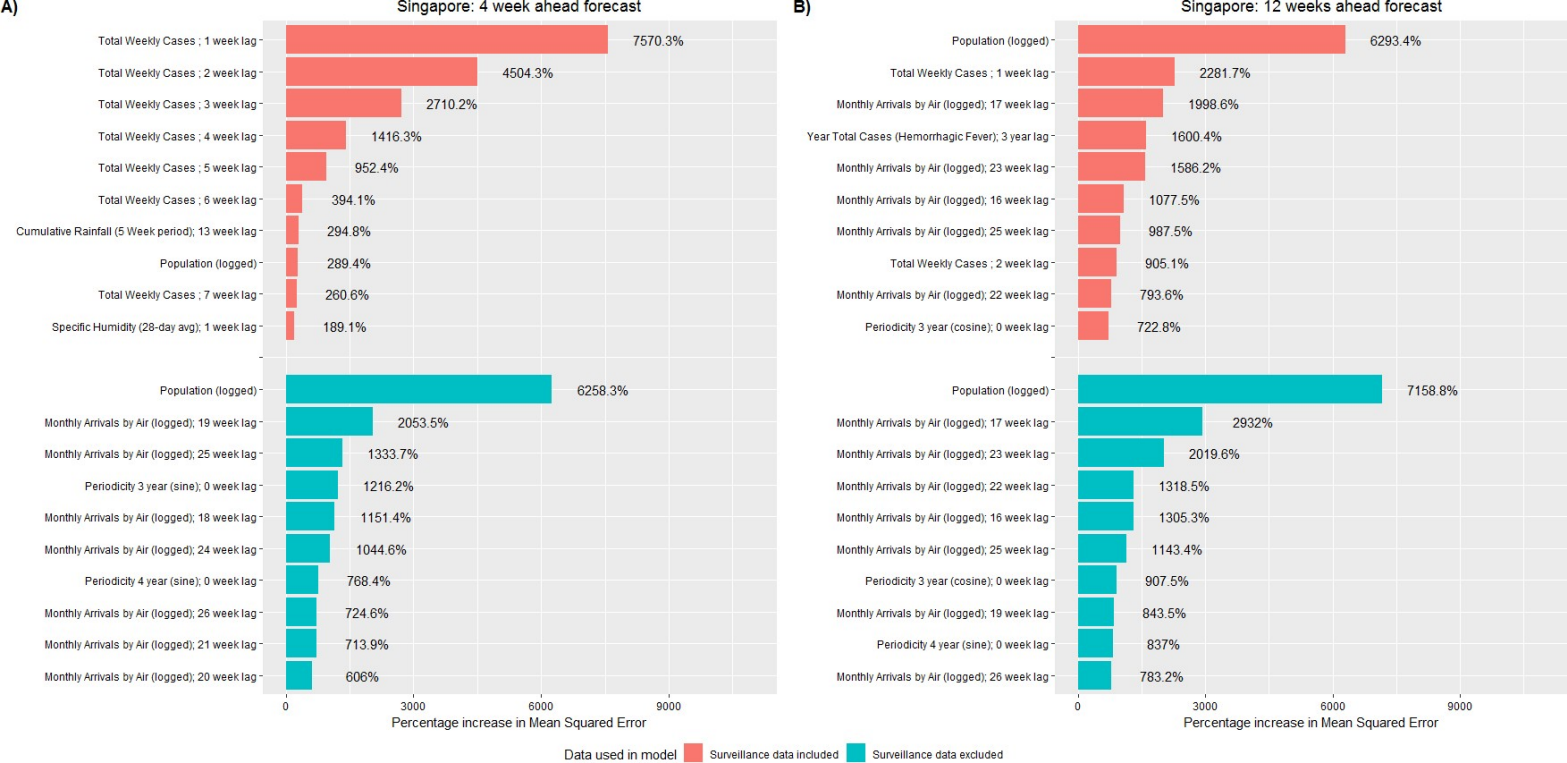
Top 10 most important predictors for the Random Forest model when predicting weekly dengue case counts, Singapore. The 10 most important predictors to the Random Forest model prior to variable reduction. Predictor importance was quantified as the percentage increase in mean squared error. Red bars indicate the model included surveillance data inputs while blue bars indicate the model did not include surveillance data inputs. Predictors are shown for forecasts made 4 (A) and 12 (B) weeks in advance.

Using variable analysis, we identified the strongest RF model predictors of weekly dengue case counts (Figs 6–8). When models included surveillance inputs, previous dengue levels were the strongest predictors for near term forecasts. When model forecasts were based upon only population, temporal, and weather data, the strongest predictors included population size, 3- and 4-year periodicity, multi-week cumulative rainfall, peak daily rainfall (Iquitos only), the average and variation in minimum daily temperature (Iquitos only), and monthly air passenger arrivals (Singapore only). Of note, these predictors were typically distributed over lag periods greater than 15 weeks. Across all study areas, we found that the inclusion of surveillance predictors had a much smaller impact on the model’s long-term forecast accuracy.

### Forecasting dengue outbreaks

Table 3 presents model MCCs, summarizing how well the optimal RF, RF-UFA, and Logistic regression models correctly predicted weekly dengue outbreaks 4 and 12 weeks in advance (results for all evaluated models are available in S7 Table). When predictions were made 4 weeks in advance and based upon surveillance, population, temporal, and weather data, both RF and RF-UFA performed worse than Logistic regression in San Juan and Sinagpore (Logistic San Juan: 0.84; Singapore: 0.57). RF-UFA had the largest MCC in Iquitos (0.56). For long-term forecasts, RF-UFA outperformed all other models where MCCs equaled 0.58, 0.61, and 0.30 in Iquitos, San Juan, and Singapore, respectively. On average, RF-UFA MCCs were 125% and 79% larger than RF and Logistic regression model MCCs.

**Table 3:** Optimal model performance when predicting weekly dengue outbreaks.

	4 weeks ahead forecast accuracy			12 weeks ahead forecast accuracy		
	Iquitos	San Juan	Singapore	Iquitos	San Juan	Singapore
	MCC	MCC	MCC	MCC	MCC	MCC
**Surveillance Data Included**						
Random Forest	0.26	0.53	0.14	0.32	0.51	0.08
Random Forest-UFA	0.56	0.67	0.27	0.58	0.61	0.30
Logistic Regression	0.44	0.84	0.57	0.57	0.60	0.09
**Surveillance Data Excluded**						
Random Forest	0.28	0.43	-0.06	0.35	0.50	0.06
Random Forest-UFA	0.49	0.66	0.22	0.58	0.61	0.27
Logistic Regression	0.38	0.53	-0.01	0.40	0.62	-0.06

Abbreviations: MCC: Matthew’s correlation coefficient.

Results for all evaluated models are available in S7 Table.

When model predictions were based upon population, temporal, and weather data only, we found that RF-UFA was the most accurate model when predicting 4 weeks ahead, (Iquitos: 0.49; San Juan: 0.66; Singapore: 0.22). For long-term predictions, RF-UFA performed best in Iquitos (MCC: 0.58) and Singapore (MCC: 0.27). While in San Juan, RF-UFA (MCC: 0.61) and Logistic regression (MCC: 0.62) had similar performance.

**Fig 9:**
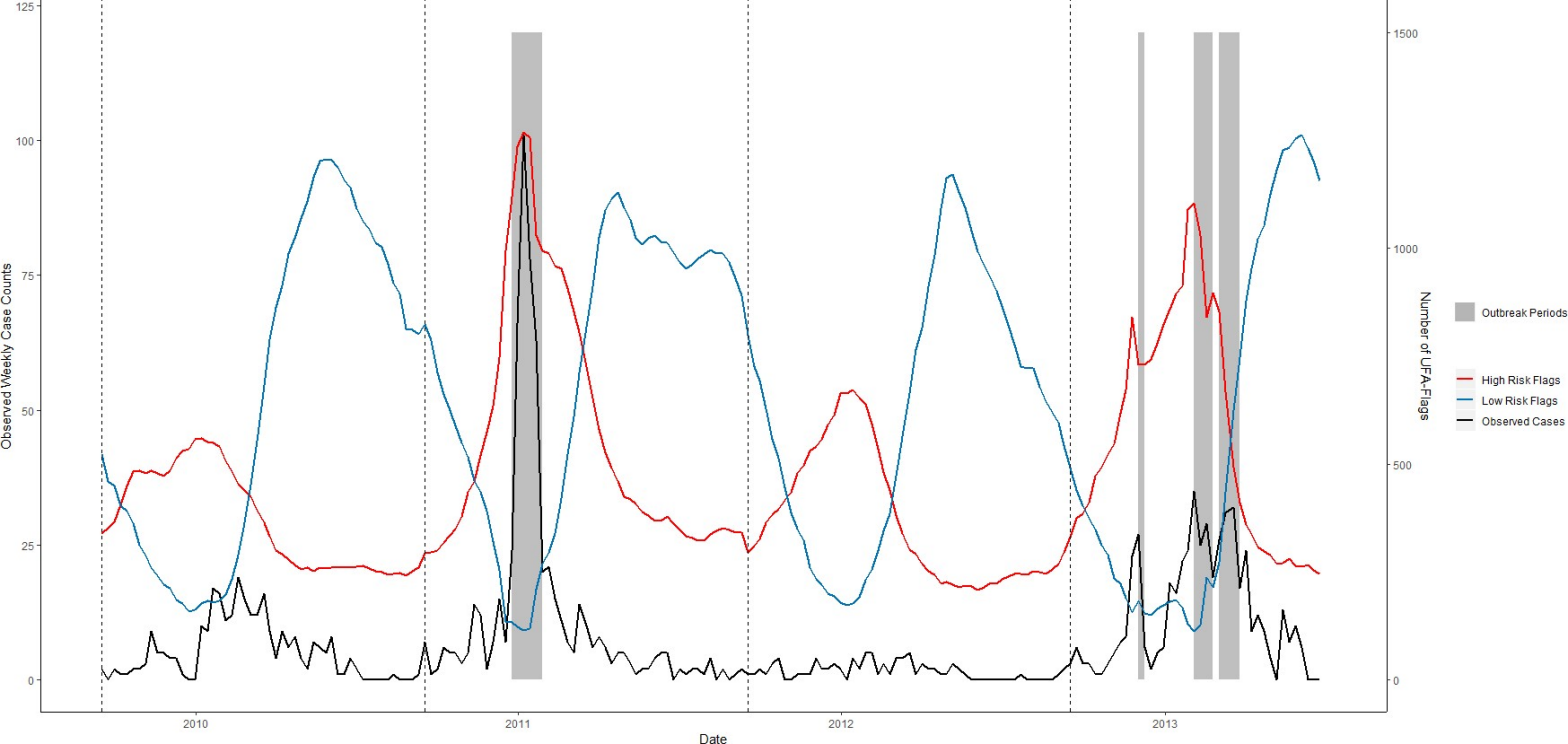
RF-UFA forecast accuracy of the temporal pattern of dengue outbreaks, Iquitos, Peru, June 2009–June 2013. The number of high-risk (red) and low-risk (blue) flags per week that are met 12 weeks in advance are plotted against weekly dengue case counts (black) in the testing data. Grey regions represent observed outbreak weeks. Thresholds were identified using UFA and are associated with dengue outbreaks 12 weeks into the future. Black dashed lines indicate the beginning of a new test set.

**Fig 10:**
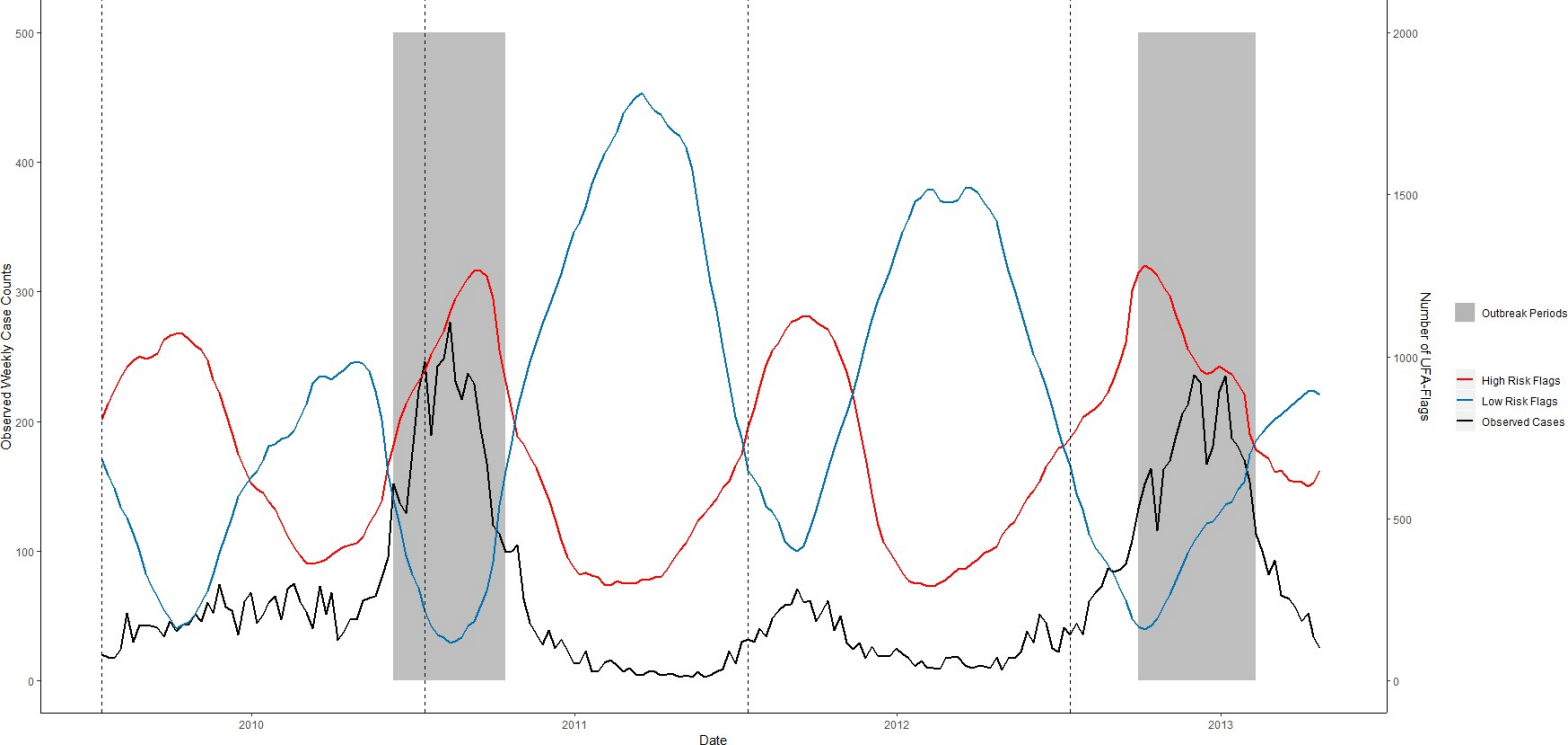
RF-UFA forecast accuracy of the temporal pattern of dengue outbreaks, San Juan, Puerto Rico, April 2009–April 2013. The number of high-risk (red) and low-risk (blue) flags per week that are met 12 weeks in advance are plotted against weekly dengue case counts (black) in the testing data. Grey regions represent observed outbreak weeks. Thresholds were identified using UFA and are associated with dengue outbreaks 12 weeks into the future. Black dashed lines indicate the beginning of a new test set.

**Fig 11:**
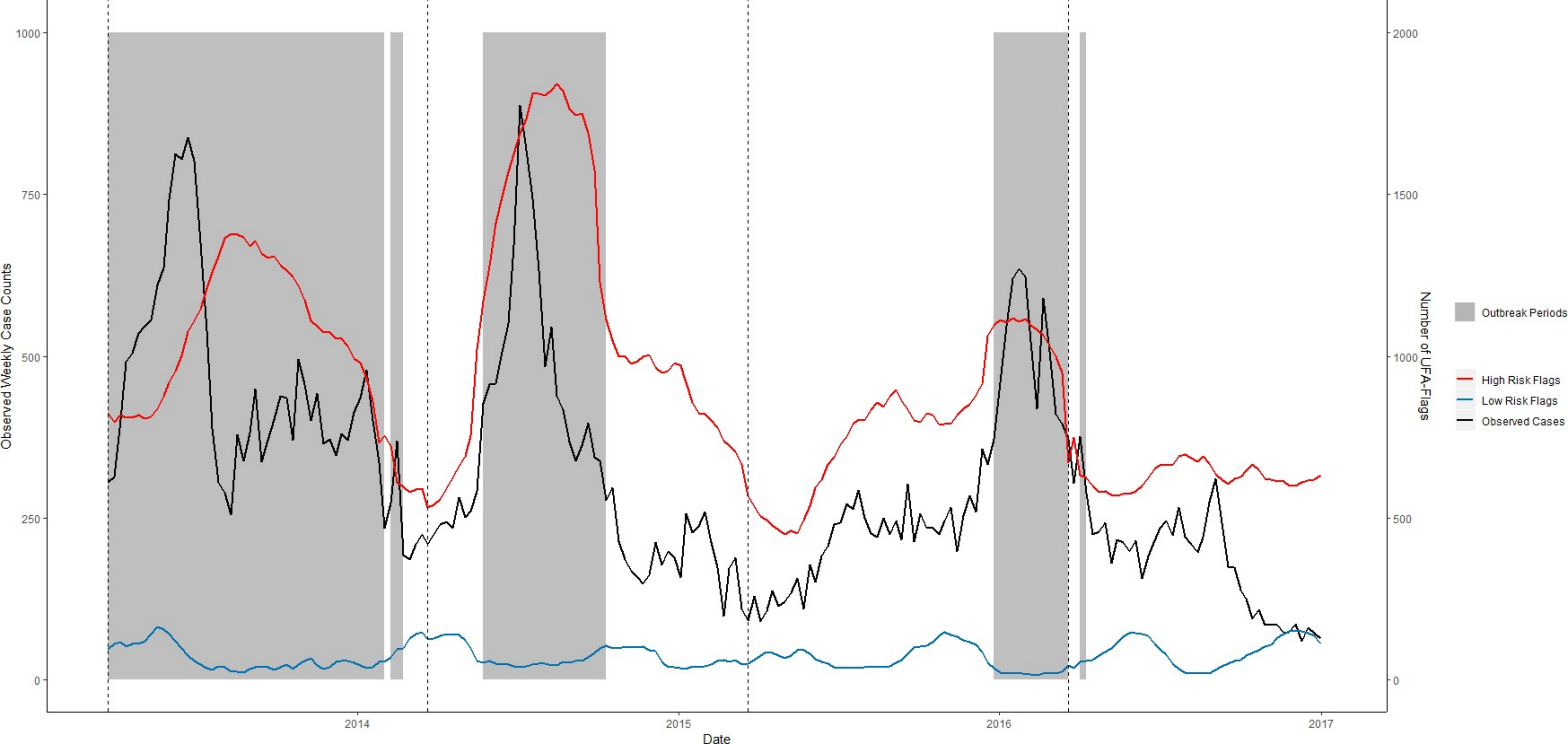
RF-UFA forecast accuracy of the temporal pattern of dengue outbreaks, Singapore, January 2013–December 2016. The number of high-risk (red) and low-risk (blue) flags per week that are met 12 weeks in advance are plotted against weekly dengue case counts (black) in the testing data. Grey regions represent observed outbreak weeks. Thresholds were identified using UFA and are associated with dengue outbreaks 12 weeks into the future. Black dashed lines indicate the beginning of a new test set.

To evaluate RF-UFA’s utility as an early warning tool, we compared the total number of high and low-risk flags per week with weekly dengue case counts (Figs 9–11). Using Pearson’s correlation, we estimated the correlation between high-risk flags and dengue cases being 0.60, 0.69 and 0.73 in Iquitos, San Juan, and Singapore. We observed a weaker and negative correlation between the number of low-risk flags and dengue cases in Iquitos (-0.35) and Singapore (-0.37), but a strong negative correlation in San Juan, (-0.79).

## Discussion

In this study, we developed RF, regression, and ARIMA models to predict dengue cases and outbreaks in 3 geographic locations. For near term forecasts, we found that RF performed better than both Poisson regression and ARIMA when the model had access to prior dengue surveillance data (Table 2). On average, RF predictions had 21% and 33% less error than Poisson regression and ARIMA models respectively. These results are consistent with other studies comparing the forecasting capabilities of RF with regression and time series models [40,45,84]. We believe that RF’s better performance is due to the model’s ability to capture the nonlinear dynamics that are part of dengue ecology [105] and to learn the trajectory of an outbreak from previously observed outbreaks. When forecasts were extended to 12 weeks in advance, the ARIMA model had the least amount of error in Iquitos and Singapore. However, in San Juan, RF performed better than Poisson regression and ARIMA. Our observation of the ARIMA model outperforming both the RF and Poisson models may be due to the ARIMA model’s ability to describe key underlying factors without being overly complex [106]. The performance of these models in providing short- and long-term forecasts appar to indicate that for short-term prediction, models benefit from an increase in complexity as the outcome is more certain and the added complexity increases model accuracy. However, for long-term predictions where the outcome is less certain, the additional model complexity appears to hurt model accuracy.

In a forecasting challenge which used similar dengue and weather data from Iquitos and San Juan; mechanistic, statistical and multimodel ensemble models were used to predict 3 dengue outcomes: peak incidence, week of peak incidence and total incidence [106]. Model performance was highly variable where models did not consistently perform well across locations and prediction targets. Similar to our study, the models did not perform well during high incidence seasons–potentially due to only having a few high incidence seasons to train the model on. Further, Johansson et al (2019) found that on average, models which included biologically meaningful data and mechanisms had lower accuracy [106]. This result appears to support our finding that ML models can at times, better leverage biologically meaningful data as they utilize a more flexible framework and do not require a priori assumptions of the predictor-target relationship.

Due to delays in case identification, current surveillance data may not be available in real time. To evaluate this limitation, we removed model inputs related to surveillance data and reassessed model performance. We found that predicted values generated by both RF and Poisson regression were similar to the general trend in dengue case counts in Iquitos and San Juan but not in Singapore. Our results show that both models were sensitive to the lack of surveillance data and model error increases. The increase in error is most likely a result of the combination of similar yearly weather patterns but high inter-annual variation in dengue spread. As such, these models are unable to fully anticipate whether or not future dengue levels will be high or low when surveillance data are unavailable.

In each study area, the random forest model had a high degree of confidence in its predictions, as evidenced by the small confidence intervals. Though the confidence intervals were small, the observed number of weekly cases were typically not included within the confidence interval. This is due to the way that the random forest model estimates the standard error: as the variation in predictions among the individual trees [102]. This result indicates that there was little variation in predicted values between individual trees.

For some scenarios, such as vector control planning, the accurate prediction of outbreak periods may prove sufficient to provide an early warning of an imminent dengue outbreak. The RF-UFA model was able to forecast weekly dengue outbreaks 12 weeks in advance where model MCCs ranged from 0.27 to 0.61 (Table 3). Further, the RF-UFA model was able to indicate periods of low dengue risk 12 weeks in advance (Figs 9–11). Of interest, RF-UFA performed well even when surveillance data inputs were removed from the model. In our analysis of the RF-UFA model we found that the number of weekly high and low-risk flags correlated well with dengue cases. Twelve weeks have been identified as the optimal lead time to enact widespread vector control efforts [11]; based upon our study results RF-UFA could be a beneficial addition to an early warning system due to its ability to identify changes in dengue spread risk.

Another study objective was to identify the strongest predictors of dengue case counts (Figs 6–8). According to our models, the strongest predictors were previous levels of dengue cases -indicating that factors such as force of infection have a stronger influence on local transmission than weather factors. These results do not imply that weather is not important but rather, once suitable weather conditions are achieved, outbreak risk becomes a function of other drivers such as: vector control, population immunity, and virus infectivity. Interestingly, in Johansson et al (2019), models which incorporated weather and surveillane data typically performed worse than models based only on surveillance data, suggesting that previous levels of dengue cases are the strongest predictors [106]. The authors further hypothesized that surveillance predictors alone may contribute equivalent information as weather predictors regarding future dengue levels and the addition of weather data may overly complicating the model [106].

For each study area, when we removed surveillance inputs from the models and predictions were based upon population, temporal and weather data only, the strongest predictors typically described multi-week weather patterns distributed over lag periods greater than 15 weeks. The observed relationships in our study are most likely due to the phase difference between seasonal signals causing the variables to become correlated rather than being related through a causal mechanistic link [57]. The strongest weather predictors demonstrated low week-to-week variation, but larger month-to-month variation. In addition, the observed lag periods are towards the maximum period by which weather variables have been observed to affect dengue spread. In Singapore, monthly air travel patterns distributed over long lag periods were also a strong predictor of dengue cases. Though global travel has been identified as an important driver of dengue outbreaks in Singapore [107], the effect of imported cases has been observed to persist a maximum of 14 to 16 weeks, suggesting that this finding is also due to phase differencing [108–112].

Our study has some limitations. Data availability may have negatively affected model performance. We could not obtain vector control data, which are critical in diminishing the size of the outbreak [11,113–115], and may confound the relationship between predictors and prediction outcomes, causing the model to learn biased predictor-outcome relationships.

To train our models, we used dengue case counts as reported by passive surveillance systems. As such, asymptomatic and clinically mild cases were most likely missed, suggesting that model predictions are underestimates of the true number of cases [2].

Our study highlighted various limitations for each modeling approach. When predicting dengue case counts, RF consistently underestimated observed extreme values, for example the 2011 outbreak in Iquitos and the 2013 outbreak in Singapore (Fig 5 and Fig 7). This consistent underestimation is a direct result of the RF’s inability to predict outside of the training set’s outcome distribution [92]. Despite this limitation, the RF model typically identified when dengue cases would peak. In contrast, Poisson regression would occasionally overestimate peak weeks with a delay, due to the model’s reliance upon the previous week’s reported cases and the linear relationship imposed by the model. When predicting weekly outbreaks, we found that all models performed poorly in Singapore, where there was an unprecedented increase in dengue cases beginning in 2013 due to a severe dengue outbreak throughout Southeast Asia [97–100]. As a result, the models were unable to account for this shift in dengue dynamics.

In evaluating RF-UFA performance, we found that this model suffered from false positives in Iquitos and San Juan. Typically, the model predicted an earlier onset and a later end to the outbreak period and, on occasion, would incorrectly predict extended outbreak periods during the traditional peak dengue months. This is certainly problematic and requires further attention since too many false positives can lead to alarm fatigue and can rapidly deplete limited resources [116].

## Conclusions

In this study, we compared the ability of ML, regression, and time-series based modeling approaches to forecast dengue case counts and outbreaks. When using dengue surveillance, population, temporal, and weather data as model inputs, RF was more accurate than both Poisson regression and ARIMA models, for near term predictions while the ARIMA model performed best for long-term predictions. We also found that when predicting dengue outbreaks, RF-UFA outperformed both RF and logistic regression models when using only population, temporal, and weather data as model inputs. Given the potential advantages of ML models the forecasting capabilities of dengue early warning systems may be improved by the inclusion of ML models.

## Supporting information

S1 Text Additional Materials and Methods. (DOCX)

S1 Table Surveillance predictor variables. (DOCX)

S2 Table Population and temporal predictor variables. (DOCX)

S3 Table Weather predictor variables. (DOCX)

S4 Table Normalized mean absolute error and mean absolute error for all evaluated Random Forest and Poisson regression models when predicting weekly dengue case counts. Abbreviations: nMAE: normalized mean absolute error; MAE: mean absolute error.
(DOCX)

S5 Table Optimal model performance when predicting weekly dengue case counts during the typical peak dengue season. *The ARIMA model was only developed using previously observed case counts. Abbreviations: nMAE: normalized mean absolute error; MAE: mean absolute error. Iquitos peak dengue season: January to July [102]. San Juan peak dengue season: May to November [104]. Singapore peak dengue season: September to February [103].
(DOCX)

S6 Table Optimal model performance when predicting weekly dengue case counts during the typical low dengue season. *The ARIMA model was only developed using previously observed case counts. Abbreviations: nMAE: normalized mean absolute error; MAE: mean absolute error. Iquitos peak dengue season: January to July [102]. San Juan peak dengue season: May to November [104]. Singapore peak dengue season: September to February [103].
(DOCX)

S7 Table Matthew’s Correlation Coefficient for each statistical modeling approach when predicting weekly outbreaks. Abbreviations: MCC: Matthew’s Correlation Coefficient.
(DOCX)

S1 Fig Description of missing weather data. S1 Fig describes the amount of missing data per variable by study area. In Iquitos and Singapore, weather stations were the primary source of missing data; for San Juan, remote sensed imagery was the most affected data source. Among all days in the data collection period, 69.5% in Iquitos, 1.1% in San Juan, and 0.6% in Singapore had at least 1 missing measurement.
(TIF)

S2 Fig 12 week forecast accuracy of the temporal pattern of dengue case counts, Iquitos, Peru, June 2009 –June 2013. Observed weekly cases counts (black area) are compared with 12 week ahead forecasts made by Random Forest and Poisson regression models. Dotted lines represent 95% confidence intervals around the model’s prediction. RF model standard errors were estimated using the infinitesimal jackknife for bagging approach [101].
(TIF)

S3 Fig ARIMA model 4 (A) and 12 (B) week forecast accuracy of the temporal pattern of dengue case counts, Iquitos, Peru, June 2009 –June 2013. Observed weekly cases counts (black area) are compared with 4 and 12 week ahead forecasts (panels A and B respectively) made by the ARIMA. Dotted lines represent 95% confidence intervals around the model’s prediction.
(TIF)

S4 Fig 12 week forecast accuracy of the temporal pattern of dengue case counts, San Juan, Puerto Rico, April 2009 –April 2013. Observed weekly cases counts (black area) are compared with 12 week ahead forecasts made by Random Forest and Poisson regression models. Dotted lines represent 95% confidence intervals around the model’s prediction. RF model standard errors were estimated using the infinitesimal jackknife for bagging approach [101].
(TIF)

S5 Fig ARIMA model 4 (A) and 12 (B) week forecast accuracy of the temporal pattern of dengue case counts, San Juan, Puerto Rico, April 2009 –April 2013. Observed weekly cases counts (black area) are compared with 4 and 12 week ahead forecasts (panels A and B respectively) made by the ARIMA. Dotted lines represent 95% confidence intervals around the model’s prediction.
(TIF)

S6 Fig 12 week forecast accuracy of the temporal pattern of dengue case counts, Singapore, January 2013 –December 2016. Observed weekly cases counts (black area) are compared with 12 week ahead forecasts made by Random Forest and Poisson regression models. Dotted lines represent 95% confidence intervals around the model’s prediction. RF model standard errors were estimated using the infinitesimal jackknife for bagging approach [101].
(TIF)

S7 Fig ARIMA model 4 (A) and 12 (B) week forecast accuracy of the temporal pattern of dengue case counts, Singapore, January 2013 –December 2016. Observed weekly cases counts (black area) are compared with 4 and 12 week ahead forecasts (panels A and B respectively) made by the ARIMA. Dotted lines represent 95% confidence intervals around the model’s prediction.
(TIF)
