# Supplementary material for: Weekly dengue forecasts in Iquitos, Peru; San Juan, Puerto Rico; and Singapore
Source: PLoS Negl Trop Dis. 2020 Oct 16;14(10):e0008710. doi: 10.1371/journal.pntd.0008710 (PMC7567393; doi:10.1371/journal.pntd.0008710)
Supplement: S1 Text — (DOCX) [file pntd.0008710.s001.docx]

# Supplemental Text S1

## Weather data limitations

In this study, we utilized a combination of sources to account for the various strengths and limitations of each weather data source. Weather stations are able to optimally represent local weather patterns, however, due to microclimates or changes in land use, weather station observations may not be generalizable to the surrounding region [8–10]. Additionally, weather stations are limited in their geographic distribution and only collect measurements on a small number of variables [8–10]. Remote sensed imagery data overcome the limited distribution of weather stations and provide accurate representations of most environmental factors [8,11,12]; however, relative to weather stations, some weather phenomenon may be inaccurately measured [8]. There is also an inherent trade-off between spatial and temporal resolutions [13,14]. Meteorological reanalysis estimates weather conditions through data assimilation –a process that relies upon empirical observations and model-based forecasts [15]. Though typically consistent with empirical measurements, these estimates can be limited by the forecasting and data assimilation methods used [16].

## Overview of machine learning models

### Random Forest

RF is an ensemble ML algorithm based upon decision trees. RF creates numerous decision trees and fits each decision tree to a bootstrapped sample of the training data. Each decision tree recursively partitions the sampled data. Each split in the decision tree represents an “if-then” decision rule for the variable used to split the data. Beginning with all sampled observations, the model randomly selects a subset of variables to split the data with. From the sampled predictors, the model identifies the decision rule that best splits the data into two groups. The best decision rule is defined as the rule that maximizes the intra-group homogeneity of the two newly created groups. The newly created subgroups are continuously split until a new decision rule does not improve the model fit or stopping criteria are met. Terminal nodes represent the predicted outcome for observations that are described by the preceding decision rules. When making a final prediction, the RF model outputs the average predicted value over all decision trees.

### Random Forest Univariate Flagging Algorithm

RF-UFA is an extension of the RF algorithm and uses the Univariate Flagging Algorithm (UFA) to transform continuous predictors into binary predictors [85]. UFA is based upon a z-score and is used to identify an optimal threshold for a single continuous predictor that is associated with a statistically significant higher (“high-risk”) or lower (“low-risk”) risk of the prediction outcome. Thresholds are selected to optimize the difference in the outcome rate for observations that fall outside of the optimal threshold and a baseline rate defined as the outcome rate within the interquartile range of the evaluated predictor [85]. Two thresholds can be identified, one above and one below the median value of the predictor. UFA can be integrated into several different prediction models; for the present study, we integrated UFA with an RF model. To do so, each statistically significant threshold (p ≤ 0.01) was used to create a binary variable (or “flag”) indicating whether or not the threshold was met for an observation [85]. The model then creates two additional variables aggregating the number of high and low-risk flags met per observation. In its application, UFA has been shown to identify thresholds that align well with subject matter expertise, while RF-UFA has been observed to perform equal to or better than other models such as RF and Logistic regression [85,86].

# References

1. Stoddard ST, Wearing HJ, Reiner Jr RC, Morrison AC, Astete H, Vilcarromero S, et al. Long-term and seasonal dynamics of dengue in Iquitos, Peru. PLoS Negl Trop Dis. 2014;8: e3003.

2. National Science and Technology Council. Epidemic Prediction Initiative. In: Dengue [Internet]. [cited 19 Apr 2017]. Available: https://predict.phiresearchlab.org/legacy/dengue/index.html

3. Forshey BM, Guevara C, Laguna-Torres VA, Cespedes M, Vargas J, Gianella A, et al. Arboviral etiologies of acute febrile illnesses in Western South America, 2000–2007. PLoS Negl Trop Dis. 2010;4: e787.

4. Sharp TM, Hunsperger E, Santiago GA, Muñoz-Jordan JL, Santiago LM, Rivera A, et al. Virus-specific differences in rates of disease during the 2010 Dengue epidemic in Puerto Rico. PLoS Negl Trop Dis. 2013;7: e2159.

5. Ong A, Goh KT. A guide on infectious diseases of public health importance in Singapore. Communicable Diseases Division, Ministry of Health [and] Communicable Disease Centre, Tan Tock Seng Hospital; 2011.

6. Althouse BM, Ng YY, Derek A. T. Cummings. Prediction of Dengue Incidence Using Search Query Surveillance. PLOS Neglected Tropical Diseases. 2011;5: e1258. doi:10.1371/journal.pntd.0001258

7. Ministry of Health Singapore. Weekly Infectious Diseases Bulletin. [cited 21 Apr 2017]. Available: https://www.moh.gov.sg/content/moh_web/home/statistics/infectiousDiseasesStatistics/weekly_infectiousdiseasesbulletin.html

8. Mendelsohn R, Kurukulasuriya P, Basist A, Kogan F, Williams C. Climate analysis with satellite versus weather station data. Climatic Change. 2007;81: 71–83.

9. Davey CA, Pielke RA. Microclimate Exposures of Surface-Based Weather Stations: Implications For The Assessment of Long-Term Temperature Trends. Bull Amer Meteor Soc. 2005;86: 497–504. doi:10.1175/BAMS-86-4-497

10. Chabot-Couture G, Nigmatulina K, Eckhoff P. An Environmental Data Set for Vector-Borne Disease Modeling and Epidemiology. PLOS ONE. 2014;9: e94741. doi:10.1371/journal.pone.0094741

11. Hay SI, Lennon JJ. Deriving meteorological variables across Africa for the study and control of vector-borne disease: a comparison of remote sensing and spatial interpolation of climate. Tropical Medicine & International Health. 1999;4: 58–71. doi:10.1046/j.1365-3156.1999.00355.x

12. Sewe MO, Tozan Y, Ahlm C, Rocklöv J. Using remote sensing environmental data to forecast malaria incidence at a rural district hospital in Western Kenya. Scientific Reports. 2017;7. Available: https://www.ncbi.nlm.nih.gov/pmc/articles/PMC5453969/

13. Kalluri S, Gilruth P, Rogers D, Szczur M. Surveillance of Arthropod Vector-Borne Infectious Diseases Using Remote Sensing Techniques: A Review. PLOS Pathogens. 2007;3: e116. doi:10.1371/journal.ppat.0030116

14. Hay SI, Tatem AJ, Graham AJ, Goetz SJ, Rogers DJ. Global Environmental Data for Mapping Infectious Disease Distribution. Adv Parasitol. 2006;62: 37–77. doi:10.1016/S0065-308X(05)62002-7

15. Parker WS. Reanalyses and observations: What’s the difference? Bulletin of the American Meteorological Society. 2016;97: 1565–1572.

16. Program (US) CCS. Reanalysis of historical climate data for key atmospheric features: Implications for attribution of causes of observed change. US Climate Change Science Program; 2008.
