# Supplementary material for: Weekly dengue forecasts in Iquitos, Peru; San Juan, Puerto Rico; and Singapore
Source: PLoS Negl Trop Dis. 2020 Oct 16;14(10):e0008710. doi: 10.1371/journal.pntd.0008710 (PMC7567393; doi:10.1371/journal.pntd.0008710)
Supplement: S1 Table — (DOCX) [file pntd.0008710.s002.docx]

S1 Table: Surveillance predictor variables.

| **Predictor variable** | **Observation Period** | **Iquitos** | **San Juan** | **Singapore** | **Lag Period** |
| --- | --- | --- | --- | --- | --- |
|  |  |  |  |  |  |
| Weekly reported dengue cases | 1 week | X | X | X | 1 to 26 weeks |
| Weekly reported dengue hemorrhagic fever cases | 1 week |  |  | X | 1 to 26 weeks |
| Cumulative dengue cases | Week 1 to week W-1 | X | X | X | 1 to 26 weeks |
| Cumulative dengue hemorrhagic fever cases | Week 1 to week W-1 |  |  | X | 1 to 26 weeks |
| Natural log of dengue annual cases | 1 year | X | X | X | 1, 2, 3, years |
| Natural log of annual dengue hemorrhagic cases | 1 year |  |  | X | 1, 2, 3, years |
