# Supplementary material for: Weekly dengue forecasts in Iquitos, Peru; San Juan, Puerto Rico; and Singapore
Source: PLoS Negl Trop Dis. 2020 Oct 16;14(10):e0008710. doi: 10.1371/journal.pntd.0008710 (PMC7567393; doi:10.1371/journal.pntd.0008710)
Supplement: S2 Table — (DOCX) [file pntd.0008710.s003.docx]

S2 Table: Population and temporal predictor variables.

| **Predictor variable** | **Observation Period** | **Iquitos** | **San Juan** | **Singapore** | **Lag Period** |
| --- | --- | --- | --- | --- | --- |
|  |  |  |  |  |  |
| Natural log of yearly population | 1 year | X | X | X | No lag |
| Natural log of monthly passenger arrivals by air | 1 month |  |  | X | 1 to 26 weeks |
| Sine periodicity | 1, 2, 3, 4 years | X | X | X | No lag |
| Cosine periodicity | 1, 2, 3, 4 years | X | X | X | No lag |
| Month | 1 month | X | X | X | No lag |
