# Supplementary material for: Weekly dengue forecasts in Iquitos, Peru; San Juan, Puerto Rico; and Singapore
Source: PLoS Negl Trop Dis. 2020 Oct 16;14(10):e0008710. doi: 10.1371/journal.pntd.0008710 (PMC7567393; doi:10.1371/journal.pntd.0008710)
Supplement: S3 Table — (DOCX) [file pntd.0008710.s004.docx]

S3 Table: Weather predictor variables.

|  |  | **Data Source** | | |  |
| --- | --- | --- | --- | --- | --- |
| **Predictor variable** | **Observation  Period** | **Weather Station** | **Remote Sensed** | **Meteorological Reanalysis** | **Lag Period** |
| Minimum air temperature (moving average) | 7-, 14-, 21-, 28-day | **X** |  | **X** | 1 to 26 weeks |
| Average air temperature (moving average) | 7-, 14-, 21-, 28-day | **X** |  | **X** | 1 to 26 weeks |
| Maximum air temperature (moving average) | 7-, 14-, 21-, 28-day | **X** |  | **X** | 1 to 26 weeks |
| Minimum air temperature (TSI scale; moving average) | 7-, 14-, 21-, 28-day | **X** |  | **X** | 1 to 26 weeks |
| Average air temperature (TSI scale; moving average) | 7-, 14-, 21-, 28-day | **X** |  | **X** | 1 to 26 weeks |
| Maximum air temperature (TSI scale; moving average) | 7-, 14-, 21-, 28-day | **X** |  | **X** | 1 to 26 weeks |
| Diurnal air temperature range (moving average) | 7-, 14-, 21-, 28-day | **X** |  | **X** | 1 to 26 weeks |
| Average surface temperature (moving average) | 7-, 14-, 21-, 28-day |  |  | **X** | 1 to 26 weeks |
| Average surface temperature (TSI scale; moving average) | 7-, 14-, 21-, 28-day |  |  | **X** | 1 to 26 weeks |
| Dew point (moving average) | 7-, 14-, 21-, 28-day |  |  | **X** | 1 to 26 weeks |
| Absolute humidity (moving average) | 7-, 14-, 21-, 28-day |  |  | **X** | 1 to 26 weeks |
| Relative humidity (moving average) | 7-, 14-, 21-, 28-day |  |  | **X** | 1 to 26 weeks |
| Specific humidity (moving average) | 7-, 14-, 21-, 28-day |  |  | **X** | 1 to 26 weeks |
| Total rainfall per rainy day (moving average) | 7-, 14-, 21-, 28-day | **X** | **X** | **X** | 1 to 26 weeks |
| Total rainfall per rainy day (Cold periods; moving average) | 7-, 14-, 21-, 28-day | **X** | **X** | **X** | 1 to 26 weeks |
| Total rainfall per rainy day (Warm periods; moving average) | 7-, 14-, 21-, 28-day | **X** | **X** | **X** | 1 to 26 weeks |
| Total rainfall per rainy day (Hot periods; moving average) | 7-, 14-, 21-, 28-day | **X** | **X** | **X** | 1 to 26 weeks |
| Total rainfall per rainy day (Cold periods; standard deviation) | 7-, 14-, 21-, 28-day | **X** | **X** | **X** | 1 to 26 weeks |
| Total rainfall per rainy day (Warm periods; standard deviation) | 7-, 14-, 21-, 28-day | **X** | **X** | **X** | 1 to 26 weeks |
| Total rainfall per rainy day (Hot periods; standard deviation) | 7-, 14-, 21-, 28-day | **X** | **X** | **X** | 1 to 26 weeks |
| Minimum air temperature (standard deviation) | 7-, 14-, 21-, 28-day | **X** |  | **X** | 1 to 26 weeks |
| Average air temperature (standard deviation) | 7-, 14-, 21-, 28-day | **X** |  | **X** | 1 to 26 weeks |
| Maximum air temperature (standard deviation) | 7-, 14-, 21-, 28-day | **X** |  | **X** | 1 to 26 weeks |
| Minimum air temperature (TSI scale; standard deviation) | 7-, 14-, 21-, 28-day | **X** |  | **X** | 1 to 26 weeks |
| Average air temperature (TSI scale; standard deviation) | 7-, 14-, 21-, 28-day | **X** |  | **X** | 1 to 26 weeks |
| Maximum air temperature (TSI scale; standard deviation) | 7-, 14-, 21-, 28-day | **X** |  | **X** | 1 to 26 weeks |
| Diurnal air temperature range (standard deviation) | 7-, 14-, 21-, 28-day | **X** |  | **X** | 1 to 26 weeks |
| Average surface temperature (standard deviation) | 7-, 14-, 21-, 28-day |  |  | **X** | 1 to 26 weeks |
| Average surface temperature (TSI scale; standard deviation) | 7-, 14-, 21-, 28-day |  |  | **X** | 1 to 26 weeks |
| Dew point (standard deviation) | 7-, 14-, 21-, 28-day |  |  | **X** | 1 to 26 weeks |
| Absolute humidity (standard deviation) | 7-, 14-, 21-, 28-day |  |  | **X** | 1 to 26 weeks |
| Relative humidity (standard deviation) | 7-, 14-, 21-, 28-day |  |  | **X** | 1 to 26 weeks |
| Specific humidity (standard deviation) | 7-, 14-, 21-, 28-day |  |  | **X** | 1 to 26 weeks |
| Total rainfall per rainy day (standard deviation) | 7-, 14-, 21-, 28-day | **X** | **X** | **X** | 1 to 26 weeks |
| Number of rainy days | 7-, 14-, 21-, 28-day | **X** | **X** | **X** | 1 to 26 weeks |
| Peak daily total rainfall | 7-, 14-, 21-, 28-day | **X** | **X** | **X** | 1 to 26 weeks |
| Total cumulative rainfall | 1-, 2-, 3-,…, 19-, 20-week | **X** | **X** | **X** | 1 to 26 weeks |
