# Supplementary material for: Weekly dengue forecasts in Iquitos, Peru; San Juan, Puerto Rico; and Singapore
Source: PLoS Negl Trop Dis. 2020 Oct 16;14(10):e0008710. doi: 10.1371/journal.pntd.0008710 (PMC7567393; doi:10.1371/journal.pntd.0008710)
Supplement: S4 Table — Abbreviations: nMAE: normalized mean absolute error; MAE: mean absolute error. (DOCX) [file pntd.0008710.s005.docx]

**S4 Table****: Normalized** **mean absolute error and mean absolute error for all evaluated Random Forest and Poisson regression models when predicting weekly dengue case counts.**

|  | **4 week ahead forecast accuracy** | | | |  | | **12 weeks ahead forecast accuracy** | | |
| --- | --- | --- | --- | --- | --- | --- | --- | --- | --- |
|  | **Iquitos** | **San Juan** | **Singapore** |  | | **Iquitos** | | **San Juan** | **Singapore** |
|  | nMAE (MAE) | nMAE (MAE) | nMAE (MAE) |  | | nMAE (MAE) | | nMAE (MAE) | nMAE (MAE) |
| **Surveillance Data Included** |  |  |  |  | |  | |  |  |
| *Random Forest* |  |  |  |  | |  | |  |  |
| Full Model | 0.87 (6.26) | 0.27 (17.53) | 0.40 (126.12) |  | | 0.99 (7.27) | | 0.51 (34.44) | 0.67 (209.74) |
| Top 1% of predictors used | 0.89 (6.46) | 0.34 (22.17) | 0.45 (140.34) |  | | 1.05 (7.69) | | 0.48 (32.46) | 0.62 (192.76) |
| Top 5% of predictors used | 0.91 (6.51) | 0.29 (18.65) | 0.41 (129.16) |  | | 1.02 (7.46) | | 0.52 (35.31) | 0.65 (202.35) |
| Top 10% of predictors used | 0.91 (6.52) | 0.28 (18.29) | 0.41 (128.37) |  | | 1.01 (7.41) | | 0.50 (33.71) | 0.68 (213.19) |
| *Poisson Regression* |  |  |  |  | |  | |  |  |
| Full Model | 1.02 (7.30) | 0.45 (29.41) | 0.51 (158.37) |  | | 0.98 (7.16) | | 0.59 (39.50) | 0.76 (239.09) |
| Top 1% of predictors used | 1.21 (8.71) | 0.49 (32.14) | 0.44 (135.98) |  | | 1.22 (8.93) | | 0.76 (51.36) | 0.66 (205.65) |
| Top 5% of predictors used | 1.19 (8.58) | 0.59 (38.30) | 0.47 (147.99) |  | | 1.06 (7.75) | | 0.73 (48.95) | 0.73 (230.08) |
| Top 10% of predictors used | 1.55 (11.15) | 0.56 (36.65) | 0.58 (182.05) |  | | 1.19 (8.76) | | 0.62 (42.05) | 0.70 (219.18) |
| **Surveillance Data Excluded** |  |  |  |  | |  | |  |  |
| *Random Forest* |  |  |  |  | |  | |  |  |
| Full Model | 0.96 (6.89) | 0.89 (38.31) | 0.65 (202.08) |  | | 0.99 (7.25) | | 0.57 (38.71) | 0.69 (214.60) |
| Top 1% of predictors used | 1.04 (7.50) | 0.65 (42.48) | 0.61 (190.00) |  | | 1.07 (7.83) | | 0.57 (38.47) | 0.62 (193.09) |
| Top 5% of predictors used | 0.97 (6.95) | 0.59 (38.80) | 0.63 (197.36) |  | | 0.96 (7.03) | | 0.60 (40.24) | 0.66 (207.30) |
| Top 10% of predictors used | 0.97 (6.95) | 0.60 (38.90) | 0.64 (199.77) |  | | 0.98 (7.21) | | 0.57 (38.46) | 0.67 (210.40) |
| *Poisson Regression* |  |  |  |  | |  | |  |  |
| Full Model | 0.88 (6.31) | 0.50 (32.63) | 0.96 (299.74) |  | | 0.87 (6.39) | | 0.59 (39.67) | 0.83 (260.33) |
| Top 1% of predictors used | 1.27 (9.11) | 0.67 (43.78) | 0.58 (181.17) |  | | 1.28 (9.37) | | 0.64 (43.15) | 0.65 (204.35) |
| Top 5% of predictors used | 1.25 (8.98) | 0.62 (40.31) | 0.76 (237.69) |  | | 0.89 (6.52) | | 0.60 (40.25) | 0.70 (220.10) |
| Top 10% of predictors used | 1.23 (8.84) | 0.53 (34.66) | 0.97 (303.53) |  | | 1.00 (7.32) | | 0.56 (37.51) | 0.74 (231.72) |

Abbreviations: nMAE: normalized mean absolute error; MAE: mean absolute error.
