# Supplementary material for: Weekly dengue forecasts in Iquitos, Peru; San Juan, Puerto Rico; and Singapore
Source: PLoS Negl Trop Dis. 2020 Oct 16;14(10):e0008710. doi: 10.1371/journal.pntd.0008710 (PMC7567393; doi:10.1371/journal.pntd.0008710)
Supplement: S5 Table — *The ARIMA model was only developed using previously observed case counts. Abbreviations: nMAE: normalized mean absolute error; MAE: mean absolute error. Iquitos peak dengue season: January to July [102]. San Juan peak dengue season: May to November [104]. Singapore peak dengue season: September to February [103]. (DOCX) [file pntd.0008710.s006.docx]

S5 Table: Optimal model performance when predicting weekly dengue case counts during the typical peak dengue season.

|  | **4 weeks ahead forecast accuracy** | | | |  | | **12 weeks ahead forecast accuracy** | | |
| --- | --- | --- | --- | --- | --- | --- | --- | --- | --- |
|  | **Iquitos** | **San Juan** | **Singapore** |  | | **Iquitos** | | **San Juan** | **Singapore** |
|  | nMAE (MAE) | nMAE (MAE) | nMAE (MAE) |  | | nMAE (MAE) | | nMAE (MAE) | nMAE (MAE) |
| **Surveillance Data Included** |  |  |  |  | |  | |  |  |
| Random Forest | 0.74 (6.83) | 0.22 (16.42) | 0.37 (106.26) |  | | 0.86 (8.03) | | 0.45 (35.92) | 0.62 (176.42) |
| Poisson Regression | 1.05 (9.68) | 0.39 (29.64) | 0.39 (109.78) |  | | 0.98 (9.09) | | 0.53 (42.04) | 0.54 (154.44) |
| ARIMA* | 0.70 (6.47) | 0.49 (37.04) | 0.56 (159.63) |  | | 0.63 (5.83) | | 0.83 (65.99) | 0.38 (106.97) |
| **Surveillance Data Excluded** |  |  |  |  | |  | |  |  |
| Random Forest | 0.80 (7.34) | 0.58 (43.74) | 0.57 (161.87) |  | | 0.84 (7.80) | | 0.55 (43.50) | 0.62 (176.27) |
| Poisson Regression | 0.89 (8.17) | 0.45 (34.14) | 0.62 (175.85) |  | | 0.77 (7.19) | | 0.51 (40.92) | 0.74 (212.13) |

*The ARIMA model was only developed using previously observed case counts.
Abbreviations: nMAE: normalized mean absolute error; MAE: mean absolute error.
Iquitos peak dengue season: January to July [103].
San Juan peak dengue season: May to November [105].
Singapore peak dengue season: September to February [104].
