# Supplementary material for: Weekly dengue forecasts in Iquitos, Peru; San Juan, Puerto Rico; and Singapore
Source: PLoS Negl Trop Dis. 2020 Oct 16;14(10):e0008710. doi: 10.1371/journal.pntd.0008710 (PMC7567393; doi:10.1371/journal.pntd.0008710)
Supplement: S6 Table — *The ARIMA model was only developed using previously observed case counts. Abbreviations: nMAE: normalized mean absolute error; MAE: mean absolute error. Iquitos peak dengue season: January to July [102]. San Juan peak dengue season: May to November [104]. Singapore peak dengue season: September to February [103]. (DOCX) [file pntd.0008710.s007.docx]

S6 Table: Optimal model performance when predicting weekly dengue case counts during the typical low dengue season.

|  | **4 weeks ahead forecast accuracy** | | | |  | | **12 weeks ahead forecast accuracy** | | |
| --- | --- | --- | --- | --- | --- | --- | --- | --- | --- |
|  | **Iquitos** | **San Juan** | **Singapore** |  | | **Iquitos** | | **San Juan** | **Singapore** |
|  | nMAE (MAE) | nMAE (MAE) | nMAE (MAE) |  | | nMAE (MAE) | | nMAE (MAE) | nMAE (MAE) |
| **Surveillance Data Included** |  |  |  |  | |  | |  |  |
| Random Forest | 1.21 (5.50) | 0.37 (19.05) | 0.43 (144.86) |  | | 1.34 (6.09) | | 0.59 (30.47) | 0.62 (211.18) |
| Poisson Regression | 0.91 (4.12) | 0.5 (26.03) | 0.43 (145.73) |  | | 1.37 (6.21) | | 0.71 (36.86) | 0.59 (200.74) |
| ARIMA* | 1.57 (7.11) | 1.01 (52.31) | 0.6 (203.77) |  | | 1.48 (6.74) | | 1.82 (94.11) | 0.43 (144.21) |
| **Surveillance Data Excluded** |  |  |  |  | |  | |  |  |
| Random Forest | 1.36 (6.16) | 0.59 (30.41) | 0.63 (213.26) |  | | 1.28 (5.84) | | 0.6 (31.26) | 0.65 (220.32) |
| Poisson Regression | 0.85 (3.84) | 0.59 (30.60) | 0.55 (186.19) |  | | 0.97 (4.41) | | 0.56 (29.22) | 0.58 (196.45) |

*The ARIMA model was only developed using previously observed case counts.
Abbreviations: nMAE: normalized mean absolute error; MAE: mean absolute error.
Iquitos peak dengue season: January to July [103].
San Juan peak dengue season: May to November [105].
Singapore peak dengue season: September to February [104].
