# Supplementary material for: Weekly dengue forecasts in Iquitos, Peru; San Juan, Puerto Rico; and Singapore
Source: PLoS Negl Trop Dis. 2020 Oct 16;14(10):e0008710. doi: 10.1371/journal.pntd.0008710 (PMC7567393; doi:10.1371/journal.pntd.0008710)
Supplement: S7 Table — Abbreviations: MCC: Matthew’s Correlation Coefficient. (DOCX) [file pntd.0008710.s008.docx]

**S****7 Table: Matthew’s Correlation Coefficient for each statistical modeling approach when predicting weekly outbreaks**

|  | **4 weeks ahead forecast accuracy** | | |  | **12 weeks ahead forecast accuracy** | | |
| --- | --- | --- | --- | --- | --- | --- | --- |
|  | **Iquitos** | **San Juan** | **Singapore** |  | **Iquitos** | **San Juan** | **Singapore** |
|  | MCC | MCC | MCC |  | MCC | MCC | MCC |
| **Surveillance Data Included** |  |  |  |  |  |  |  |
| *Random Forest* |  |  |  |  |  |  |  |
| Full Model | 0.26 | 0.53 | 0.14 |  | 0.24 | 0.51 | -0.06 |
| Top 1% of predictors used | 0.38 | 0.60 | 0.32 |  | 0.32 | 0.44 | 0.08 |
| Top 5% of predictors used | 0.36 | 0.59 | 0.39 |  | 0.32 | 0.51 | 0.06 |
| Top 10% of predictors used | 0.31 | 0.59 | 0.34 |  | 0.26 | 0.51 | -0.01 |
| *Random Forest-UFA* |  |  |  |  |  |  |  |
| Full Model | 0.64 | 0.67 | 0.27 |  | 0.51 | 0.61 | 0.23 |
| Top 1% of predictors used | 0.57 | 0.76 | 0.27 |  | 0.58 | 0.61 | 0.21 |
| Top 5% of predictors used | 0.56 | 0.72 | 0.30 |  | 0.56 | 0.61 | 0.30 |
| Top 10% of predictors used | 0.56 | 0.68 | 0.28 |  | 0.55 | 0.61 | 0.29 |
| *Logistic Regression* |  |  |  |  |  |  |  |
| Full Model | 0.47 | 0.85 | 0.60 |  | 0.39 | 0.55 | -0.01 |
| Top 1% of predictors used | 0.4 | 0.84 | 0.73 |  | 0.57 | 0.55 | 0.09 |
| Top 5% of predictors used | 0.44 | 0.86 | 0.57 |  | 0.36 | 0.57 | -0.06 |
| Top 10% of predictors used | 0.46 | 0.86 | 0.60 |  | 0.35 | 0.60 | 0.02 |
| **Surveillance Data Excluded** |  |  |  |  |  |  |  |
| *Random Forest* |  |  |  |  |  |  |  |
| Full Model | 0.28 | 0.48 | -0.06 |  | 0.24 | 0.50 | -0.06 |
| Top 1% of predictors used | 0.28 | 0.43 | 0.04 |  | 0.32 | 0.43 | -0.03 |
| Top 5% of predictors used | 0.36 | 0.48 | -0.03 |  | 0.35 | 0.50 | -0.06 |
| Top 10% of predictors used | 0.30 | 0.48 | -0.02 |  | 0.26 | 0.49 | 0.06 |
| *Random Forest-UFA* |  |  |  |  |  |  |  |
| Full Model | *0.62* | 0.66 | 0.23 |  | 0.50 | 0.60 | 0.27 |
| Top 1% of predictors used | 0.50 | 0.67 | 0.33 |  | 0.53 | 0.57 | 0.23 |
| Top 5% of predictors used | 0.49 | 0.67 | 0.22 |  | 0.58 | 0.59 | 0.25 |
| Top 10% of predictors used | 0.56 | 0.66 | 0.24 |  | 0.52 | 0.61 | 0.21 |
| *Logistic Regression* |  |  |  |  |  |  |  |
| Full Model | 0.39 | 0.53 | 0.02 |  | 0.39 | 0.55 | -0.06 |
| Top 1% of predictors used | 0.42 | 0.58 | 0.17 |  | 0.40 | 0.53 | -0.06 |
| Top 5% of predictors used | 0.40 | 0.59 | 0.02 |  | 0.37 | 0.62 | -0.06 |
| Top 10% of predictors used | 0.38 | 0.61 | -0.01 |  | 0.35 | 0.57 | -0.06 |

Abbreviations: MCC: Matthew’s Correlation Coefficient
