# Supplementary figures and images for: Weekly dengue forecasts in Iquitos, Peru; San Juan, Puerto Rico; and Singapore
Source: PLoS Negl Trop Dis. 2020 Oct 16;14(10):e0008710. doi: 10.1371/journal.pntd.0008710 (PMC7567393; doi:10.1371/journal.pntd.0008710)

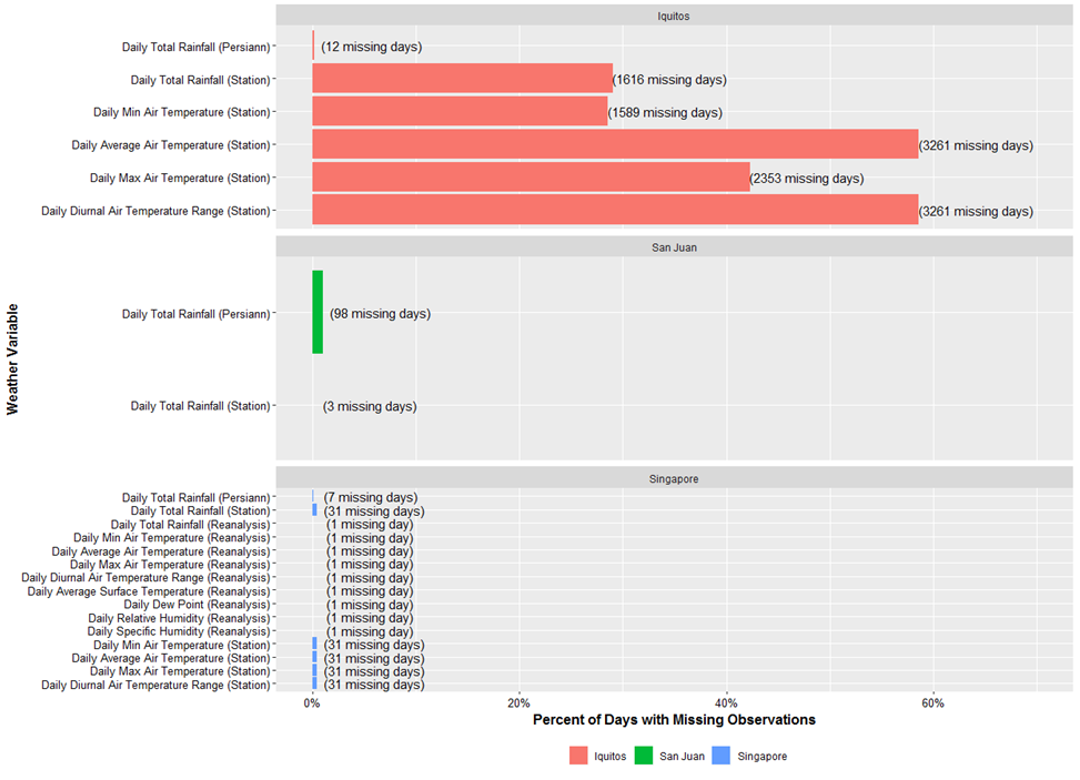

Supplement: S1 Fig — describes the amount of missing data per variable by study area. In Iquitos and Singapore, weather stations were the primary source of missing data; for San Juan, remote sensed imagery was the most affected data source. Among all days in the data collection period, 69.5% in Iquitos, 1.1% in San Juan, and 0.6% in Singapore had at least 1 missing measurement. (TIF) [file pntd.0008710.s009.tif]

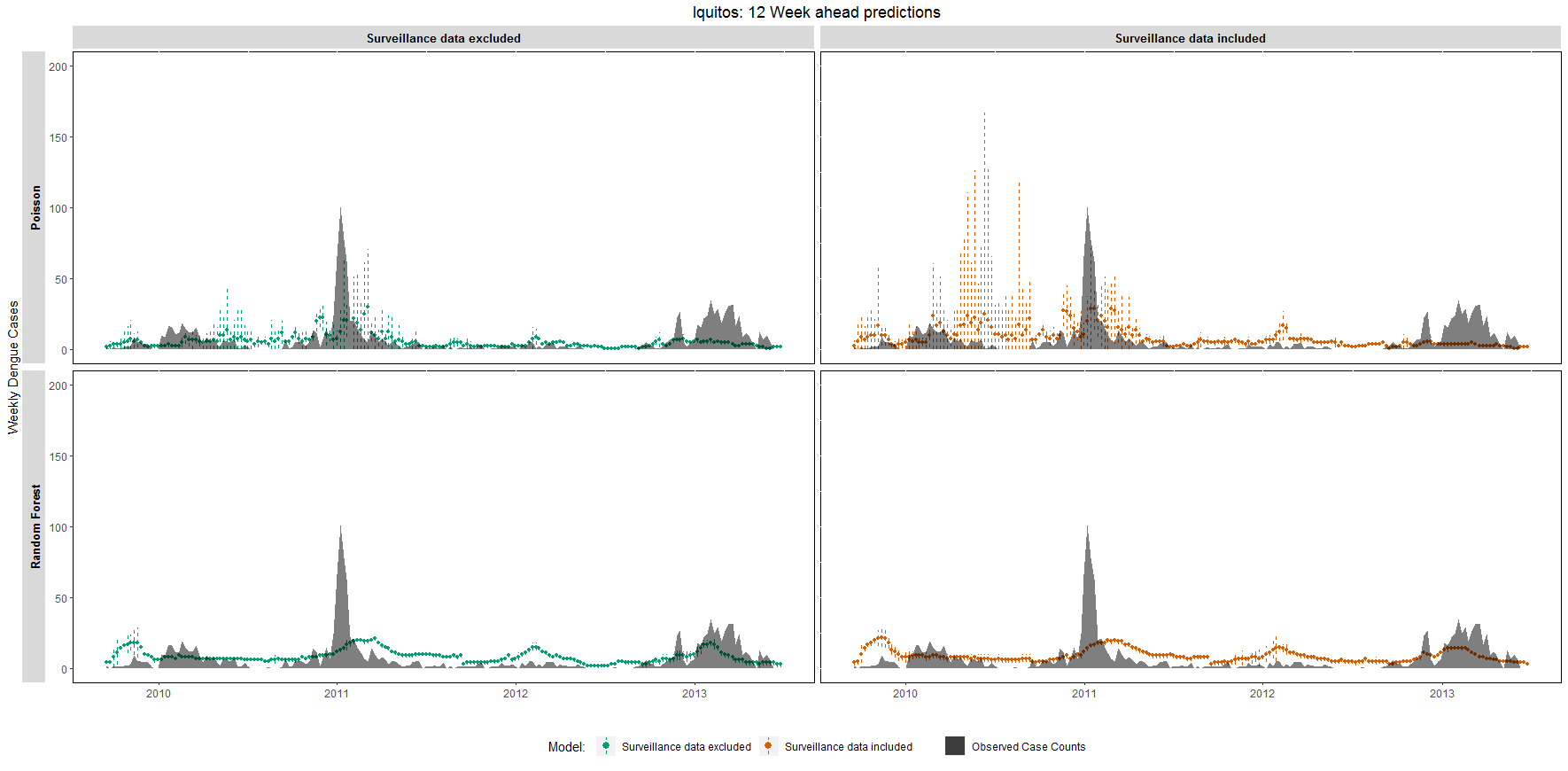

Supplement: S2 Fig — Observed weekly cases counts (black area) are compared with 12 week ahead forecasts made by Random Forest and Poisson regression models. Dotted lines represent 95% confidence intervals around the model’s prediction. RF model standard errors were estimated using the infinitesimal jackknife for bagging approach [101]. (TIF) [file pntd.0008710.s010.tif]

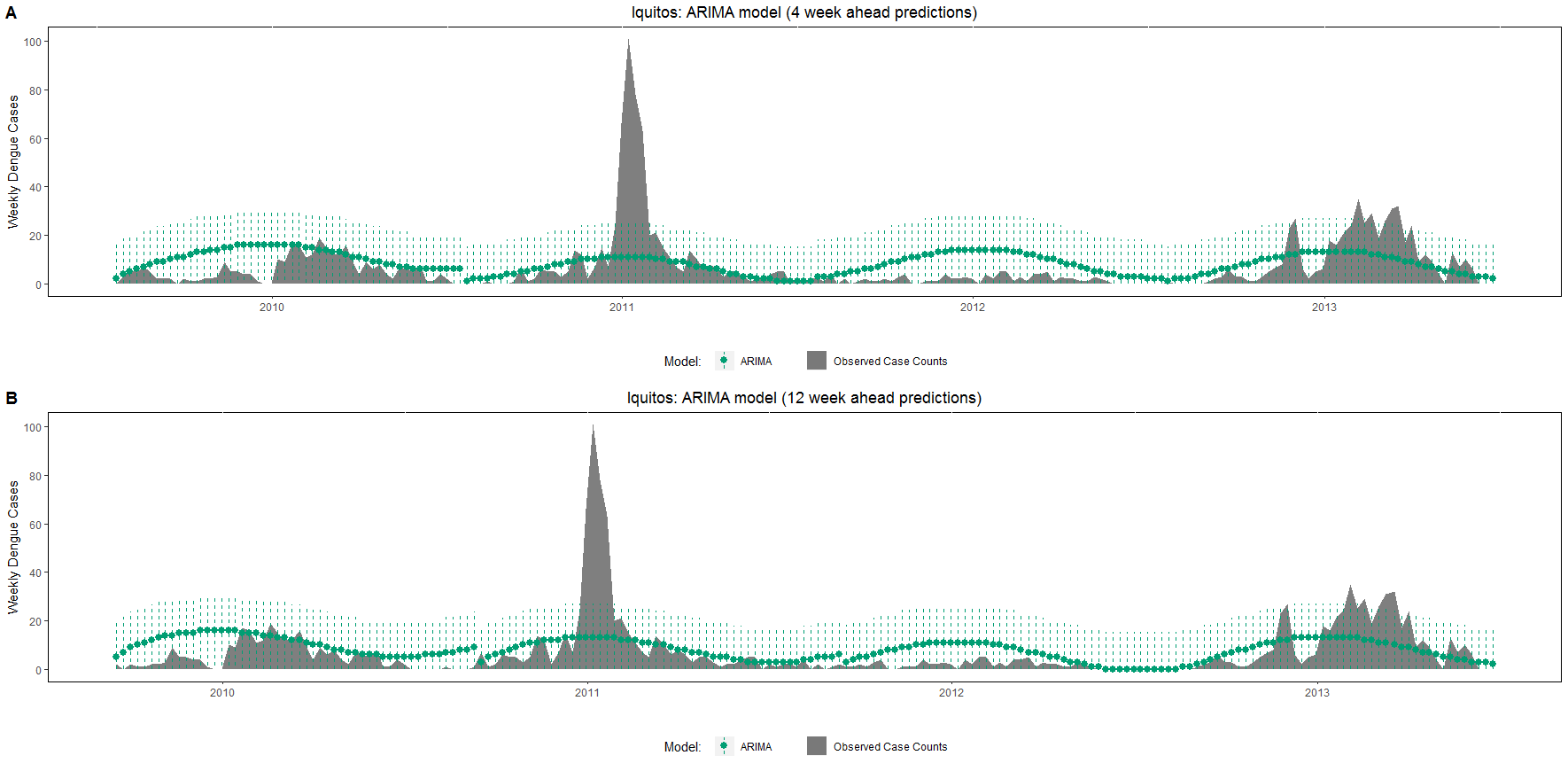

Supplement: S3 Fig — ARIMA model 4 (A) and 12 (B) week forecast accuracy of the temporal pattern of dengue case counts, Iquitos, Peru, June 2009 –June 2013. Observed weekly cases counts (black area) are compared with 4 and 12 week ahead forecasts (panels A and B respectively) made by the ARIMA. Dotted lines represent 95% confidence intervals around the model’s prediction. (TIF) [file pntd.0008710.s011.tif]

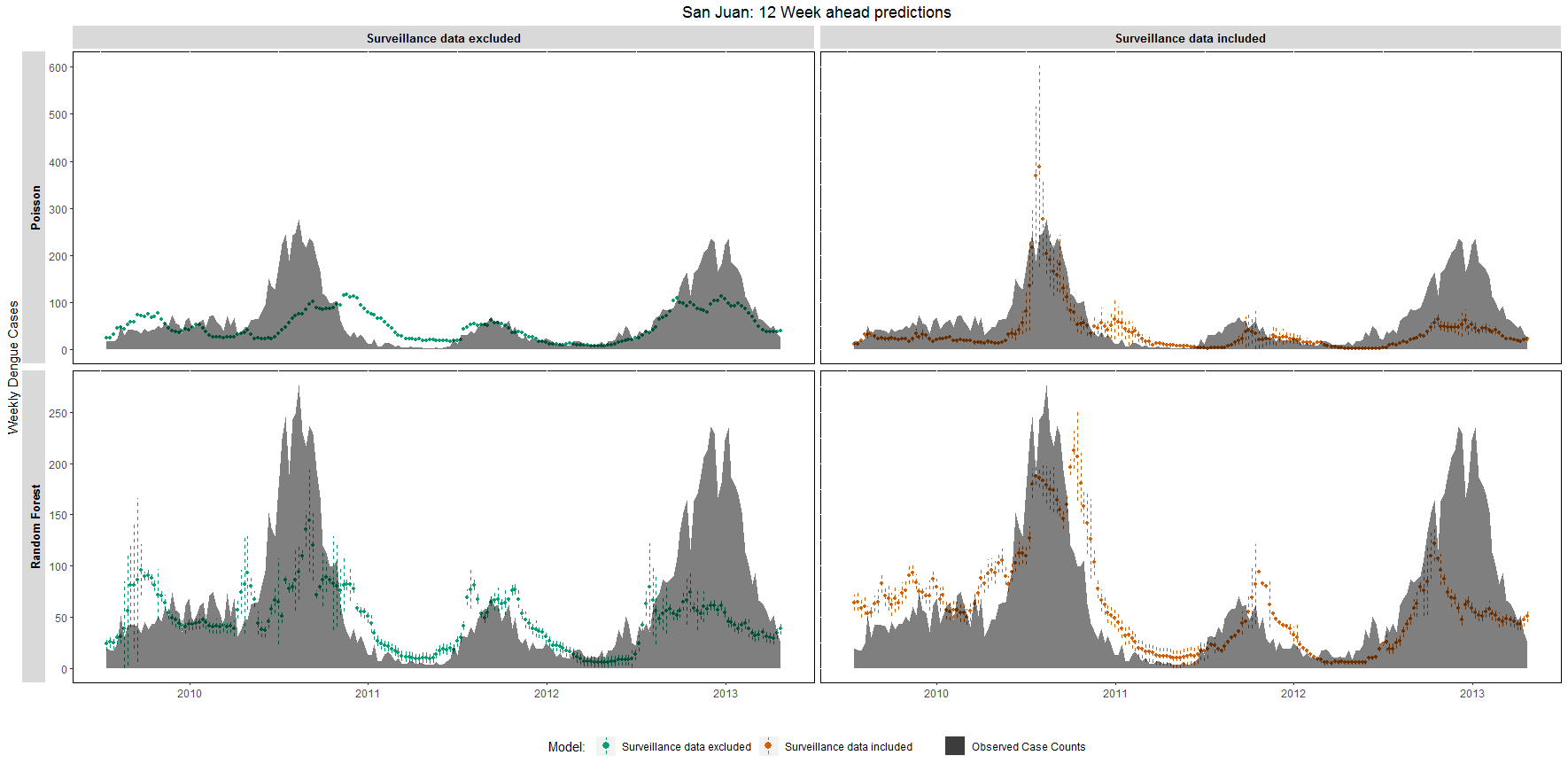

Supplement: S4 Fig — Observed weekly cases counts (black area) are compared with 12 week ahead forecasts made by Random Forest and Poisson regression models. Dotted lines represent 95% confidence intervals around the model’s prediction. RF model standard errors were estimated using the infinitesimal jackknife for bagging approach [101]. (TIF) [file pntd.0008710.s012.tif]

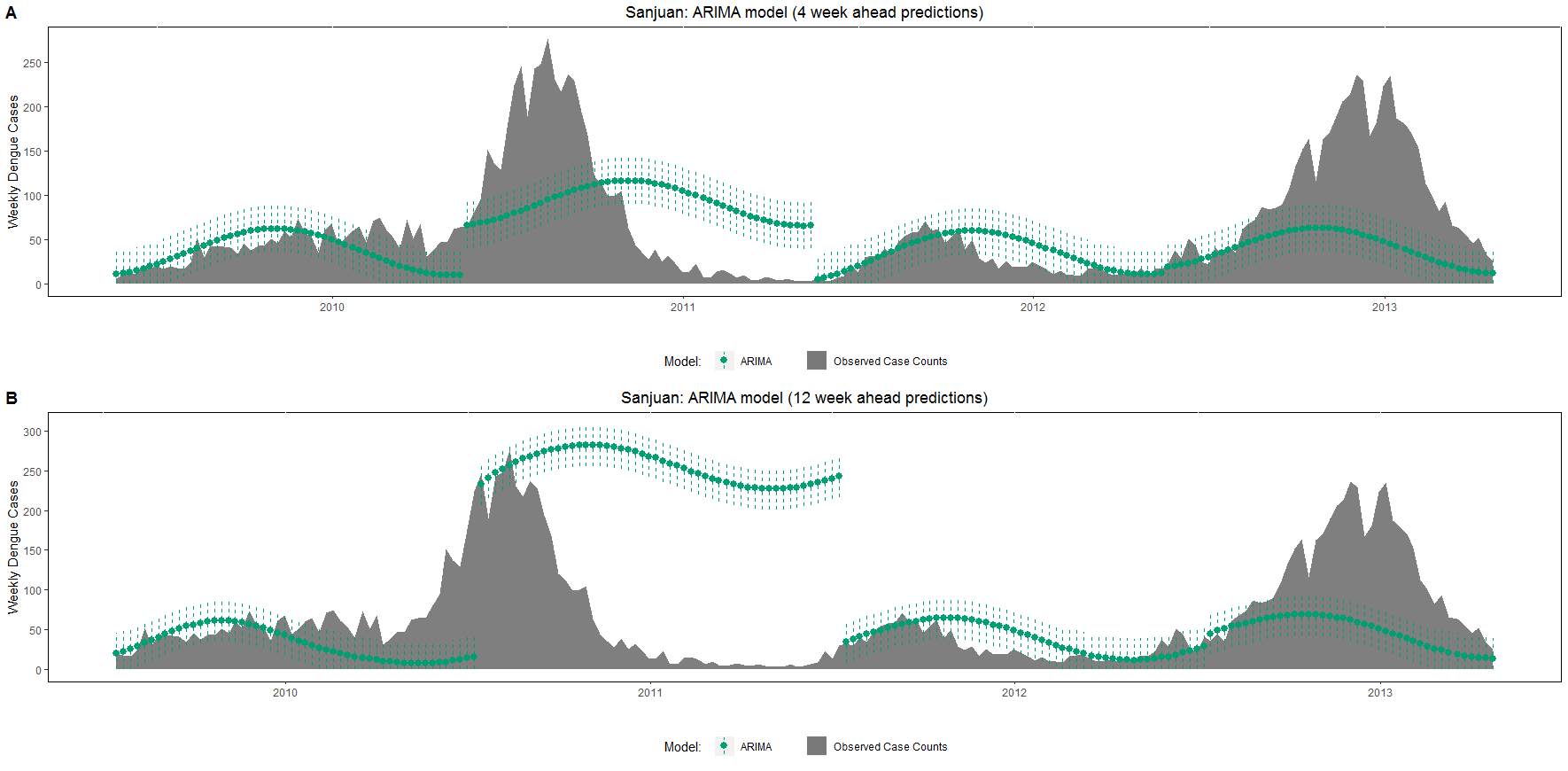

Supplement: S5 Fig — ARIMA model 4 (A) and 12 (B) week forecast accuracy of the temporal pattern of dengue case counts, San Juan, Puerto Rico, April 2009 –April 2013. Observed weekly cases counts (black area) are compared with 4 and 12 week ahead forecasts (panels A and B respectively) made by the ARIMA. Dotted lines represent 95% confidence intervals around the model’s prediction. (TIF) [file pntd.0008710.s013.tif]

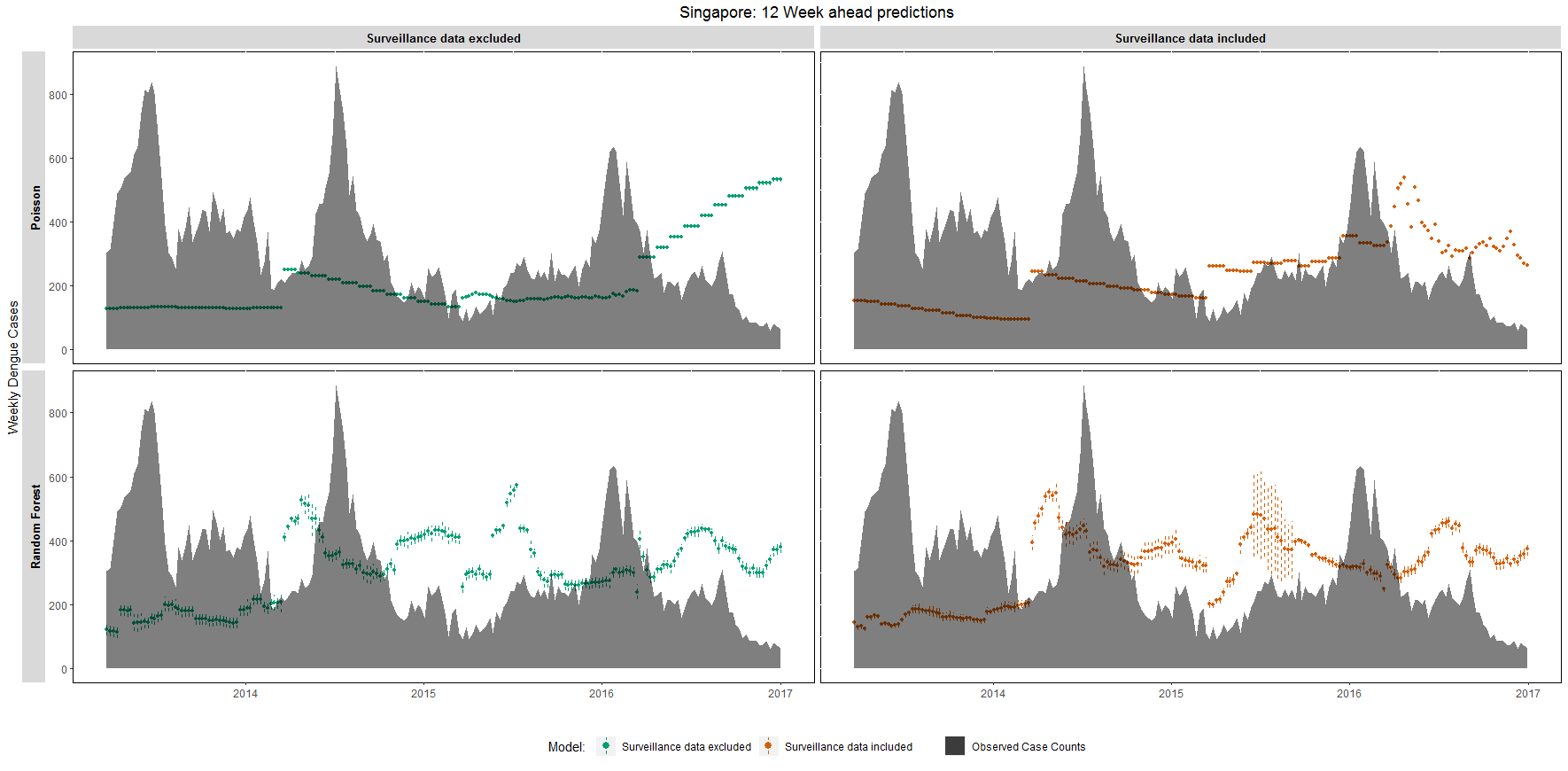

Supplement: S6 Fig — Observed weekly cases counts (black area) are compared with 12 week ahead forecasts made by Random Forest and Poisson regression models. Dotted lines represent 95% confidence intervals around the model’s prediction. RF model standard errors were estimated using the infinitesimal jackknife for bagging approach [101]. (TIF) [file pntd.0008710.s014.tif]

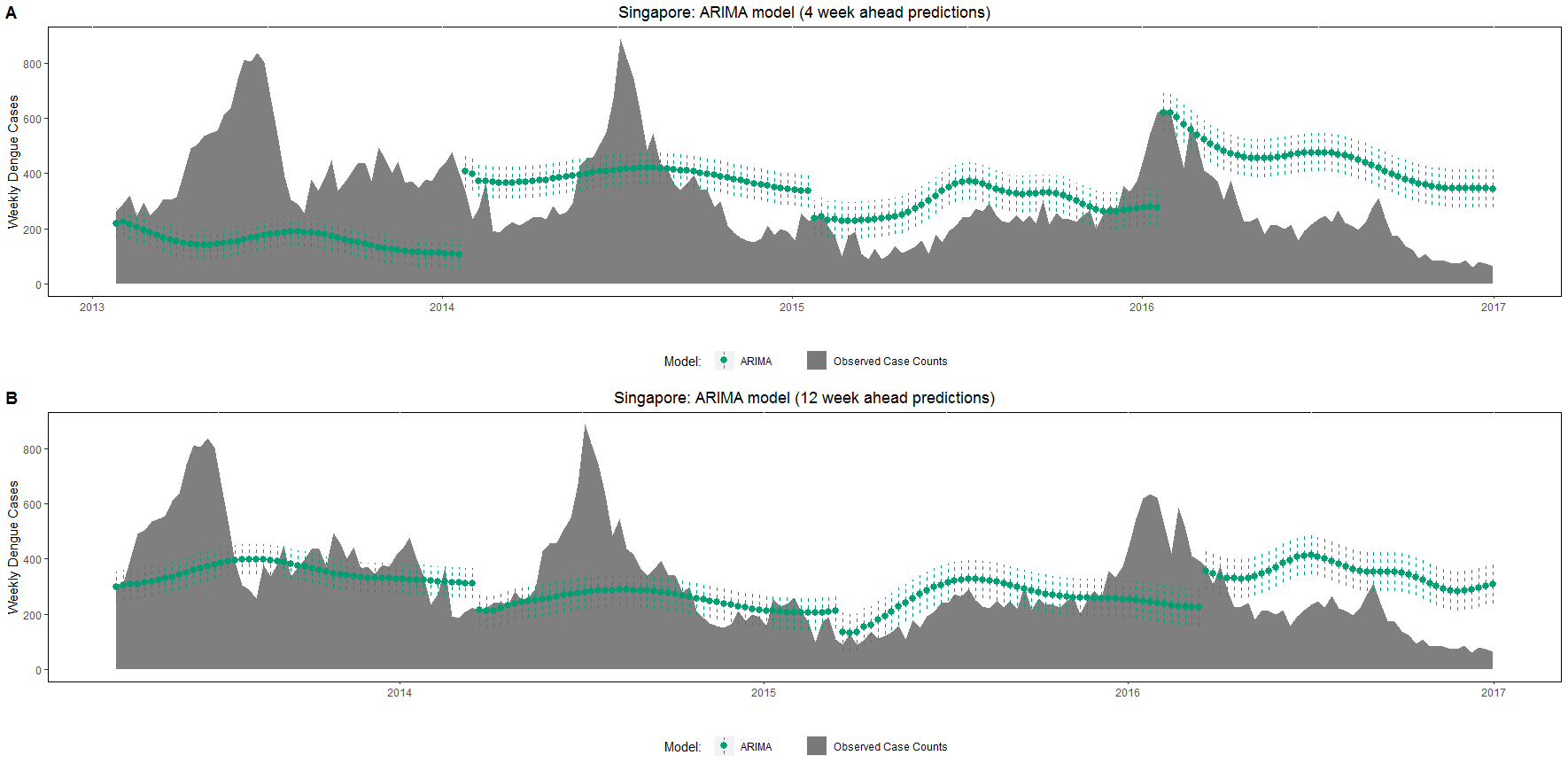

Supplement: S7 Fig — ARIMA model 4 (A) and 12 (B) week forecast accuracy of the temporal pattern of dengue case counts, Singapore, January 2013 –December 2016. Observed weekly cases counts (black area) are compared with 4 and 12 week ahead forecasts (panels A and B respectively) made by the ARIMA. Dotted lines represent 95% confidence intervals around the model’s prediction. (TIF) [file pntd.0008710.s015.tif]
